# Rising performance-efficiency demands in female junior tennis: A longitudinal analysis of ITF World Tennis Tour Juniors rankings, 2004–2025

**DOI:** 10.1371/journal.pone.0359538

**Published:** 2026-09-29

**Authors:** Michal Bozděch

**Affiliations:** Department of Physical Education and Social Sciences, Faculty of Sports Studies, Masaryk University, Brno, Czechia; University of Illinois Urbana-Champaign, UNITED STATES OF AMERICA

## Abstract

Long-term changes in ranking-point production efficiency among female players on the ITF World Tennis Tour Juniors were examined between 2004 and 2025. Year-end ranking data comprised 8,510 player-year observations from 5,919 players aged 12–18 years. Elite status was defined annually using a two-stage data-driven knee-point procedure: players were ordered according to Total Ranking Points (TRP), the knee percentile was identified from the smoothed Total Singles Points profile across this TRP-based ordering, and the threshold was applied to the same percentile scale. Performance efficiency was assessed using Singles PPE, Doubles PPE, and Total PPE, with Total PPE defined as TRP divided by total competitive event exposure. In pooled descriptives excluding 2020–2021, median Singles PPE was 64.62 in elite versus 26.56 in non-elite players; corresponding values were 48.57 versus 29.00 for Doubles PPE and 40.38 versus 17.71 for Total PPE. Positive Year × elite-status interactions were identified in linear player-year models adjusted for age, discipline-specific competitive event exposure, COVID-disrupted seasons, and repeated player observations, corresponding to additional average annual increases of 10.81%, 8.97%, and 9.25%, respectively. However, non-COVID natural-spline models showed that temporal differentiation was non-linear and did not support uniform monotonic widening across 2004–2025. In lagged sensitivity analyses, prior-year elite status was associated with higher subsequent PPE, but Year × prior-year elite-status interactions were not significant in the strict non-COVID specification. Forecasts based on 20 non-COVID annual elite medians projected higher PPE through 2030 under continuation of the observed linear trend, while event-exposure scenarios indicated fewer competitive event exposures associated with fixed TRP benchmarks at higher projected efficiency. These projections should be interpreted as conditional ranking-system scenarios rather than measures of physiological load or individual participation requirements. Overall, structurally defined elite players were characterised by persistently higher ranking-point production efficiency and significantly different, non-linear historical trajectories.

## Introduction

### Junior tennis as a ranking-driven developmental pathway

The International Tennis Federation (ITF) World Tennis Tour Juniors is established as the fundamental framework structuring the developmental pathway for elite youth athletes [1,2]. Within this highly organized system, junior rankings are utilized not merely as a descriptive measure of on-court performance but as a critical mechanism for selection and career advancement. Access to high-tier competitive opportunities, such as Grand Slam events, as well as the allocation of national federation funding and player visibility among scouts and professional academies, are structurally determined by an individual’s position within the ranking hierarchy [2,3]. Recent evidence, however, suggests that the predictive validity of these rankings for long-term career success is increasingly questioned, as successful juniors and successful seniors have been identified as largely two disparate populations. Systematic meta-analytical findings reveal that at the international championship level, there is only a 7.2% overlap between elite U17/18 juniors and elite seniors, indicating that these groups are 92.8% disparate. This lack of continuity is further evidenced by the fact that 89.2% of international-level juniors fail to reach an equivalent senior level, while 82.0% of successful seniors never achieved international status during their late junior years. In elite female tennis specifically, while 58.75% of top junior participants successfully transition to the professional WTA circuit, only 24.58% achieve a top 500 ranking, underscoring that the junior-to-senior transition is a complex, non-linear developmental phase where juvenile performance marks often fail to account for long-term potential [3–5].

Furthermore, achieving elite status within the junior ranks is widely regarded as a primary prerequisite for the subsequent junior-to-senior transition, serving as a gateway to the professional circuit [1]. This transition is identified as a normative turning phase in career development where athletes must cope with escalating demands to ensure continued progression. Participation in elite international junior tournaments remains a significant indicator of career potential, as players with such experience frequently achieve earlier breakthroughs into the professional Top 300 compared to their peers [4]. Given the dynamic and highly competitive nature of this pathway, the systematic analysis of long-term shifts in ranking-based performance is necessitated to comprehend how competitive standards and performance requirements have evolved. Therefore, evaluating these systemic patterns over an extended period is essential for identifying the historical development of elite standards and providing a multidimensional perspective on the factors shaping athletic progression in the female youth game [1].

### Performance efficiency as a complementary construct

Conceptually, performance efficiency was defined as the ratio between ranking outcomes achieved and competitive opportunities used, expressed as ranking points collected per unit of competitive event exposure. This construct was operationalised through the Points per Event (PPE) metric, which reflects ranking-point production efficiency rather than the mere accumulation of ranking points through tournament participation. PPE was not used as a replacement for Total Ranking Points (TRP), but as a complementary ranking-derived indicator that provides an exposure-adjusted perspective on player performance within the ITF junior ranking system. Because PPE remains based on ranking points, it should not be interpreted as a direct measure of intrinsic sporting ability; rather, it captures how efficiently players converted competitive exposure into ranking value [2,6]. This distinction is practically relevant because reliance on cumulative ranking points alone may obscure how efficiently players convert competitive exposure into ranking value. However, PPE should not be interpreted as a direct indicator of physiological load, injury risk, or burnout, because the present dataset does not include match duration, training exposure, travel burden, recovery status, or injury outcomes [4].

Theoretical anchoring for this multifaceted approach is provided by distinguishing between Singles PPE, Doubles PPE, and Total PPE, thereby allowing for a comprehensive evaluation of efficiency across different competitive formats of the game. Metrics grounded in efficiency, such as PPE or technical effectiveness, have been demonstrated to explain a substantial portion of the variance in competitive performance and offer high predictive validity for future professional standing [1,6]. For example, top-tier professional performance is increasingly characterized by extreme efficiency outliers, with elite players generating up to four times more points per match than the average professional cohort [4]. Consequently, the inclusion of efficiency-based indicators is necessitated to provide a more nuanced understanding of elite standards within the highly structured pathway of junior tennis [2].

### Need for structural rather than arbitrary elite classification

Within the domain of talent identification, elite status is frequently defined through the use of arbitrary performance percentiles or fixed ranking positions, such as a Top 100 cutoff or a static 10% threshold [7]. However, it is argued that such predetermined benchmarks may fail to account for the shifting competitive density and the non-linear structure of the ranking system over extended periods [2]. The reliance on these arbitrary snapshots is further complicated by evidence that successful juniors and successful seniors represent largely two disparate populations, with a mere 7.2% overlap identified at the international championship level [4,5]. Consequently, arbitrary junior thresholds may inadvertently exclude the vast majority of future professional elites who do not reach these fixed milestones during adolescence [4,5].

To address these limitations and reduce the subjectivity inherent in manual classification, a structural rather than arbitrary approach to defining elite status is necessitated. Furthermore, the application of inferentially robust techniques to estimate these cut-off values allows for a more precise tracking of long-term shifts in performance standards, particularly in an environment characterized by systemic disruptions and point-table reforms. By distinguishing players located within the structurally dominant segment of the annual TRP distribution, this framework provides a data-driven basis for evaluating ranking-point production efficiency and its longitudinal development within the ITF junior ranking environment [5].

### Temporal change and performance inflation in junior tennis

Within the dynamic environment of the ITF World Tennis Tour Juniors, it is recognized that the structural elements of the ranking system—including tournament availability and point-table distributions—are subject to significant temporal shifts. It is posited that as the systemic landscape and competitive density evolve, the functional benchmarks for elite performance must be re-evaluated [8]. This necessity is underscored by recent data indicating a substantial intensification of competition; while the pool of ranked professional players has increased by only 8% since 2019, the junior population has expanded by 30%, suggesting that the pathway toward professional entry has become increasingly constrained [8]. This constraint occurs alongside a broader trend of performance stagnation in absolute athletic marks, suggesting that as athletes approach biological and physiological limits, the separation between cohorts is increasingly defined by minute efficiency gains rather than massive improvements in raw power [9].

Within this context, the concept of “performance-efficiency inflation” is introduced to describe the escalating standards of point production required to sustain elite status. It is argued that rising efficiency demands are placed upon athletes, whereby achieving structurally derived milestones necessitates a more proficient conversion of competitive opportunities into ranking outcomes than in previous decades. As the game evolves into a more explosive and dynamic sport, these escalating standards are increasingly linked to superior physical markers, such as serve velocity and change-of-direction (CoD) proficiency, which have become more significant predictors of ranking than in earlier eras [10,11]. Consequently, the systematic analysis of long-term trajectories is necessitated to determine whether an efficiency gap has widened between elite and non-elite segments over time. Furthermore, to ensure the robustness of these observed trends, the implementation of adjusted player-year models is required to account for confounding developmental factors such as age, competitive event exposure, and the impact of systemic disruptions.

### Developmental context: age and female junior tennis

Within the ITF World Tennis Tour Juniors, age is recognized as a fundamental contextual factor shaping the performance landscape, particularly for female players aged 12–18 who undergo intensive biological and psychological development [3,11–13]. It is posited that within this specific developmental window, even minor disparities in chronological age—ranging from six months to one year—may correspond to substantial differences in physical maturity, competitive experience, and technical stability [14]. This period is further complicated by the Relative Age Effect (RAE), where players born earlier in the selection year often benefit from temporary physical advantages that do not necessarily translate into long-term professional proficiency [4,5]. In the female category, biological maturation often accelerates prior to age 13, potentially providing temporary performance advantages that differentiate players of similar chronological ages [14]. While intensive physical development typically begins to stabilize between the ages of 16 and 18, this period remains a critical turning point for achieving elite status and navigating the junior-to-senior transition [15]. Evidence from elite athletic populations indicates that women generally achieve their peak competitive performance earlier than men, particularly in technical and explosive disciplines, which places unique temporal pressure on the late-junior developmental phase [4,16]. In the context of this study, age is interpreted as a developmental framework through which performance must be evaluated, rather than as an isolated explanatory variable. Consequently, a systematic evaluation of the age profile of elite and non-elite players is necessitated to comprehend how structural elite status is associated with developmental maturity.

### Forecasting and practical translation into event-exposure scenarios

Beyond the retrospective characterisation of historical trends, prospective scenario modelling may help to contextualise how elite ranking-efficiency benchmarks could evolve in the near future. In a ranking-driven developmental pathway, descriptive historical evidence alone may be insufficient for interpreting whether current performance standards are likely to remain stable or continue to intensify. Forecasting was therefore included not as an attempt to identify an optimal predictive algorithm or to generate deterministic predictions, but as a transparent continuation-of-trend framework for examining the possible future trajectory of elite ranking-point production efficiency [17].

The practical relevance of such projections lies in their translation into event-exposure scenarios. By relating projected Total PPE values to fixed TRP benchmarks, it becomes possible to estimate how many competitive event exposures would theoretically be associated with selected ranking targets under different levels of ranking-point production efficiency. This approach provides a ranking-system scenario analysis rather than a direct measure of competitive burden, physiological workload, travel exposure, or athlete health risk. Nevertheless, when interpreted cautiously, event-exposure scenarios may offer a useful supplementary reference for coaches, federations, and player-development programmes by illustrating whether ranking progression is more likely to be achieved through higher efficiency, greater competitive exposure, or a combination of both [1,15,18].

### Aim and research questions

Two closely related publications using ITF World Tennis Tour Juniors year-end ranking archives provide the immediate empirical background to the present analysis. A previous study of female junior players examined data from 2004–2024 at the population-threshold level, estimating annual P90, P75, and P50 cut-offs for TRP and aggregated PPE, analysing age profiles and geographic representation, and forecasting threshold trajectories through 2029 using Bayesian Prophet models [2]. A separate study of male junior players over 2004–2024 used a related system-level framework to examine TRP and PPE percentile thresholds, ranking inflation, developmental and geographic characteristics, and Bayesian forecasts through 2029 [19]. The present study therefore shares an underlying public ITF data source and substantial calendar-year overlap with these publications, particularly with the previous female analysis, but addresses a different unit of inference and research question. Rather than re-estimating population-level percentile thresholds or geographic equity, the current analysis extends the female observational series through 2025 and focuses on within-population differentiation between annually and structurally defined elite and non-elite players. The specific analytical framework comprises separate Singles PPE, Doubles PPE, and Total PPE trajectories; cluster-robust player-year models; year-normalised, lagged, COVID-period, and flexible-year sensitivity analyses; and conditional 2026–2030 efficiency and event-exposure scenarios. These player-level group comparisons and sensitivity analyses were not part of the previous publications.

Against this background, the present study was designed to examine the historical development, adjusted temporal trajectories, and conditional event-exposure scenarios of ranking-point production efficiency among structurally defined elite and non-elite female players on the ITF World Tennis Tour Juniors between 2004 and 2025. The primary analytical question was whether ranking-point production efficiency, expressed through Singles PPE, Doubles PPE, and Total PPE, developed differently over time between the two structurally defined groups. Because both contemporaneous elite-status classification and PPE were derived from ranking-point information, the primary analyses were designed to evaluate longitudinal differentiation within the ranking system rather than an association between fully independent classification and outcome constructs; this dependence was additionally examined through the lagged sensitivity analysis described in the Methods. Adjusted models were implemented to account for age, competitive event exposure, COVID-period disruption, and repeated player observations, while flexible-year modelling was used to evaluate whether the temporal differentiation depended on a linear functional form. The relationship between structural elite status and age-related developmental profiles was additionally evaluated as contextual evidence. Beyond the characterisation of historical patterns, the study aimed to project elite PPE values for 2026–2030 and to translate these projections into conditional event-exposure scenarios linked to selected TRP benchmarks. The study therefore extends previous system-level threshold research by examining how ranking-point production efficiency is differentiated within the female junior population and how this differentiation develops under alternative longitudinal specifications.

## Materials and methods

### Study design, population, and data acquisition

This study employed a retrospective, observational design based on secondary analysis of publicly available ranking data from international junior tennis. The target population comprised female junior tennis players competing on the International Tennis Federation (ITF) World Tennis Tour Juniors circuit. All analyses were restricted to female players in order to ensure conceptual homogeneity and to avoid conflation with sex-specific developmental and competitive dynamics.

Ranking data were obtained from the official ITF website under the World Tennis Tour Junior Rankings section. Specifically, year-end ranking datasets were collected for each season from 2004 through 2025. Year-end rankings were selected to ensure temporal comparability across seasons and to reflect cumulative competitive performance within each calendar year. A retrospective audit of the source dataset identified substantial heterogeneity in the depth of the available ranking coverage. For female players, the non-COVID annual source datasets generally contained approximately 82–116 records and extended to a correspondingly similar ranking position, whereas the 2020 and 2021 source datasets contained 3,514 and 3,137 records and extended to ITF ranking positions 3,297 and 2,935, respectively. The source coverage returned to 100 records, extending to rank 100, in 2022. The available project metadata do not permit a definitive retrospective distinction between differences in the depth of the historical ITF archive display and differences in the depth of the original data extraction. Accordingly, the markedly larger 2020–2021 samples are treated as a source-coverage characteristic of these seasons rather than as evidence of an equivalent expansion in the underlying competitive population. The resulting dataset represents a longitudinal cross-sectional series spanning 22 competitive seasons. The raw data included player identifiers, year-end Total Ranking Points (TRP), discipline-specific ranking points for singles and doubles, and discipline-specific counts of competitive participation. For the present analyses, these participation counts were operationalised as competitive event entries for singles and doubles, with total competitive event exposure defined as the sum of singles and doubles entries. Based on these variables, performance-efficiency (PPE) metrics were computed as ranking points per competitive event exposure for singles (PPE_Singles), doubles (PPE_Doubles), and total performance (PPE_Total), following the operational definitions specified below. PPE variables therefore represent derived efficiency indicators computed from publicly available source data rather than directly observed measures. Inclusion criteria were applied at the discipline level. For the Singles analysis, observations with zero singles competitive event entries within a given year (n = 3) were excluded. Similarly, for the Doubles analysis, observations with zero doubles competitive event entries (n = 128) were excluded. These exclusions were implemented because performance efficiency cannot be meaningfully defined in the absence of competitive participation within a given discipline. By contrast, no observations were excluded from the Total efficiency analysis, as each player necessarily recorded at least one competitive event entry in either singles or doubles within each year. As a result, discipline-specific sample sizes differed slightly across Singles, Doubles, and Total analyses, while the underlying player population remained consistent.

### Ethics statement

This study used retrospective secondary data obtained from publicly accessible ITF World Tennis Tour Juniors ranking archives. No participants were recruited, no intervention was performed, and no direct contact with players occurred. The data were accessed for research purposes between 20 and 31 January 2026.

The source records consisted of publicly available sporting performance and ranking information. The dataset did not include health data, contact details, training-load data, injury records, or any non-public personal information. Where publicly available player identifiers were present in the source records, they were used only for data organisation, record linkage, and avoidance of duplicate player-year entries. The analytical focus was placed on aggregated player-year patterns, and all results are reported in aggregated form.

Because the study was based exclusively on publicly accessible secondary ranking data, involved no direct interaction with athletes, and did not analyse sensitive or private personal information, formal ethics approval and informed consent were not required. For data sharing, publicly available player identifiers were replaced with stable anonymised player identifiers to minimise potential privacy risks while preserving longitudinal linkage across seasons. The anonymised analysis-ready dataset underlying the reported findings is provided in the Supplementary Materials (S1 File), and the complete author-generated R analysis code used to reproduce the reported analyses is provided as S2 File.

### Data processing and statistical analysis

The analytical dataset was prepared from the cleaned female ITF World Tennis Tour Juniors year-end ranking data. The unit of analysis was the player-year observation. Only observations from players aged 12–18 years were retained for the main analyses. This resulted in an analytical sample of 8,510 player-year observations from 5,919 unique female junior players across the period 2004–2025.

Elite status was operationalised using an annual two-stage data-driven knee-point procedure applied separately within each competitive year. First, players were ordered according to Total Ranking Points (TRP), and a TRP-based percentile position was calculated, with lower percentile values representing stronger ranking positions. Second, the structural knee percentile was identified from the smoothed profile of Total Singles Points across this TRP-based percentile ordering. The resulting annual knee percentile was then applied to the same TRP-based percentile scale: players whose percentile position was less than or equal to the annual knee percentile were classified as elite, whereas all remaining players were classified as non-elite. The knee point represented the location of greatest structural change in the smoothed Singles Points profile across the TRP-based ordering. This procedure allowed the elite boundary to vary according to the empirical structure observed within each competitive year rather than imposing a fixed ranking or percentile threshold across the study period. The annual knee percentiles, sample sizes, numbers of players classified as elite and non-elite, and classification-consistency checks are reported in the Supplementary Materials (S1 Table). Because the knee was estimated from a within-year percentile distribution, substantial differences in annual ranking-list depth could affect both percentile resolution and the shape of the smoothed Singles Points profile from which the knee was identified. The 2020–2021 knee thresholds were therefore not assumed to be directly comparable to those derived from substantially shallower annual lists; these seasons were retained for descriptive classification but excluded from the primary annual trend models. Performance efficiency was examined using discipline-specific and total points-per-event indicators. Singles PPE was calculated as total singles points divided by the number of singles competitive event entries, and Doubles PPE as total doubles points divided by the number of doubles competitive event entries. Total PPE was calculated as total ranking points divided by total competitive event exposure, defined as the sum of singles and doubles competitive event entries. Throughout the manuscript, competitive event entry therefore denotes the unit contributing to the PPE denominator, whereas tournament denotes the underlying organised tournament as a distinct competitive setting. Because a player may record both a singles and a doubles entry within the same tournament, total competitive event exposure does not necessarily correspond to the number of unique tournaments entered. Total PPE was used to represent ranking-point production efficiency in relation to the official total ranking-point score. Because elite status was derived from annual ranking-point information and the PPE indicators included ranking points in their numerator, elite-status classification and contemporaneous PPE outcomes were not fully independent. Accordingly, the primary analyses were interpreted as examining ranking-point production efficiency among structurally defined high-ranking-point players, rather than as testing an entirely independent latent performance construct. To examine the extent to which the observed group differences persisted after temporally separating classification from outcome, an additional lagged sensitivity analysis linked elite status in year t − 1 to PPE in year t among players observed in consecutive seasons. Prior to modelling, repeated player-year keys within the lagged sample were audited. Thirty duplicated keys with identical model-relevant values were reduced to a single record, whereas one duplicated key containing discrepant PPE and exposure values was excluded rather than resolved arbitrarily. Separate cluster-robust models were then fitted for Singles PPE, Doubles PPE, and Total PPE using log1p-transformed PPE as the outcome and adjusting for year, age, and discipline-specific competitive event exposure; models retaining COVID-disrupted outcome years additionally included a binary COVID-period covariate. Models including a Year × prior-year elite-status interaction were used to test whether the prospective PPE difference changed over historical time. Because the exceptionally deep 2020–2021 source-data coverage also affected the number of available consecutive-year observations, three specifications were examined: all available consecutive years, exclusion of outcome years 2020–2021, and a strict non-COVID specification excluding outcome years 2020–2022 so that neither the outcome nor its immediately preceding elite-status classification originated from a COVID-disrupted season. In the strict non-COVID specification, models without the interaction term were additionally fitted to estimate the overall association between prior-year elite status and subsequent PPE. Full lagged sensitivity results are reported in S10 Table. Descriptive statistics were first calculated separately for elite and non-elite players. Because the distributions of PPE indicators were right-skewed, medians and interquartile ranges were treated as the primary descriptive statistics. Means, standard deviations, minimum values and maximum values were also computed to describe the full distribution and to capture upper-tail performance behaviour. Because the exceptionally deep source-data coverage in 2020–2021 caused these two seasons to dominate pooled full-period summaries, the primary pooled descriptive statistics were calculated after excluding 2020–2021, consistent with the primary annual trend analyses. Full-period pooled descriptive statistics including all seasons are provided separately in S9 Table for transparency. To examine longitudinal development, the dataset was transformed into a long format with three performance-efficiency indicators: Singles PPE, Doubles PPE and Total PPE. Annual descriptive summaries were calculated for each combination of year, elite status and PPE indicator. These summaries included the number of observations, number of players, mean, standard deviation, median, interquartile range, minimum and maximum values. Annual differences between elite and non-elite players were also calculated using both median differences and median ratios.

The years 2020 and 2021 were identified as COVID-disrupted seasons and were additionally characterised by substantially deeper ranking coverage in the source dataset than the surrounding years. The female analytical sample comprised 3,514 player-year observations in 2020 and 3,127 in 2021, compared with approximately 82–116 observations in the non-COVID annual datasets. Audit procedures confirmed that this imbalance was already present in the source data and was not generated by subsequent analytical filtering; no exact duplicate rows were identified, and the comparatively small number of repeated player-year keys could not account for the difference in annual sample size. These seasons were retained in descriptive visualisations of the full annual trajectories but were excluded from the primary models of annual median trends because both the COVID-related competitive disruption and the exceptional source-data coverage reduced their comparability with the remaining seasons. Accordingly, descriptive annual trajectories were presented for the full 2004–2025 period, whereas primary trend estimation based on annual medians was conducted using non-COVID years only. To evaluate the robustness of the annual median trend models to alternative treatments of the COVID-disrupted period, sensitivity analyses were conducted using three specifications: all years included, exclusion of 2020–2021, and exclusion of 2020–2022; robust regression models were additionally fitted as a complementary check against influential annual values.

Linear models were fitted to annual median PPE values to evaluate whether the development of performance efficiency differed between elite and non-elite players. Separate models were estimated for Singles PPE, Doubles PPE and Total PPE. In each model, annual median PPE was entered as the dependent variable, year was centred at 2004, elite status was entered as a categorical predictor, and the interaction between year and elite status was included to estimate whether the annual change in PPE differed between the two groups. The non-elite group was used as the reference category. The interaction term was therefore interpreted as the additional annual change in median PPE among elite players relative to non-elite players.

To evaluate whether the observed longitudinal separation between elite and non-elite players remained evident after accounting for individual-level covariates, adjusted player-year models were subsequently fitted. Because PPE indicators were strongly right-skewed, each dependent variable was transformed using the log1p transformation, defined as log(PPE + 1). Separate models were estimated for Singles PPE, Doubles PPE and Total PPE. The general model structure was: log1p(PPE) = Year × Elite status + Age + competitive event exposure + COVID-disrupted year.

Year was centred at 2004, elite status was entered as a categorical predictor, and the non-elite group was used as the reference category. Age was included as a developmental covariate. Competitive event exposure was adjusted using the discipline-specific exposure variable: the number of singles competitive event entries for Singles PPE, the number of doubles competitive event entries for Doubles PPE, and total competitive event exposure for Total PPE. COVID-disrupted years were included as a binary covariate in these player-year models. Cluster-robust standard errors, clustered at the player level, were used to account for repeated observations of the same players across seasons. The primary linear interaction term represented the additional average annual change in log-transformed PPE among elite players relative to non-elite players after adjustment for age, competitive event exposure, and COVID-disrupted years. For interpretability, interaction coefficients were also back-transformed and expressed as approximate percentage changes using 100 × [exp(b) − 1].

Because a linear effect of year on the log1p scale implies an approximately exponential trajectory after back-transformation, an additional flexible-year sensitivity analysis was conducted to assess whether the temporal differentiation between elite and non-elite players depended on this functional-form assumption. Natural cubic splines with three degrees of freedom were fitted for centred year and interacted with elite status in otherwise equivalent player-year models. The COVID-disrupted seasons 2020–2021 were excluded from these spline models because their exceptionally deep source-data coverage could disproportionately influence the estimated flexible trajectory. Joint cluster-robust Wald tests were used to evaluate the spline-by-elite-status interaction across the three spline terms. Adjusted predictions from these non-COVID spline models were generated on the back-transformed log1p scale. Predictions were generated at the median covariate values of the corresponding model samples: age 16 years and competitive event exposure of 8 singles competitive event entries, 7 doubles competitive event entries, and 15 total competitive event exposures, respectively. The linear interaction estimates were retained as parsimonious summaries of the average temporal trend, whereas the spline models were used to evaluate the shape of the trajectories and to avoid interpreting the back-transformed linear specification as a literal year-specific growth curve. As a sensitivity analysis addressing potential historical changes in the ranking-point environment, PPE outcomes were additionally normalised within each competitive year. For Singles PPE, Doubles PPE and Total PPE, two year-normalised indicators were calculated: a within-year z-score of log1p(PPE) and a within-year PPE percentile. These indicators expressed each player’s efficiency relative to the distribution of players observed in the same year. Cluster-robust linear models were then refitted using the year-normalised PPE indicators as outcomes, with the same substantive focus on the Year × elite-status interaction.

A contextual age analysis was conducted to determine whether differences in performance efficiency could be partly attributable to developmental age. Age distributions were summarised by elite status using means, standard deviations, medians and interquartile ranges. A simple linear model was used to estimate the overall age difference between elite and non-elite players. Annual age trajectories were then examined using linear models with year, elite status and their interaction as predictors. Because the COVID-disrupted years could distort longitudinal age patterns, a sensitivity model was also fitted after excluding 2020–2021. This age analysis was treated as contextual rather than as a primary outcome analysis, because age was already included as an adjustment variable in the player-year PPE models.

To estimate prospective developments in performance-efficiency requirements among structurally defined elite players, forecasting models were fitted to annual median PPE values among elite players. Forecasting was conducted separately for Singles PPE, Doubles PPE, and Total PPE. Consistent with the preceding trend analyses, the COVID-disrupted years 2020–2021 were excluded from model estimation. The forecasting input therefore consisted of elite annual median PPE values from 2004–2019 and 2022–2025, providing 20 annual observations for each PPE series. For each PPE indicator, a linear model was fitted with annual median PPE as the dependent variable and centred year as the predictor. More flexible time-series models, including ARIMA specifications, were not selected because the forecasting objective was descriptive continuation of the observed historical trend rather than optimisation of predictive performance. With only 20 annual observations per series, reliable identification, selection, and validation of autoregressive and moving-average structure would have been limited and would have introduced additional model-selection uncertainty. The linear specification was therefore retained as a parsimonious, transparent, and directly interpretable representation of the non-COVID historical trend. Forecasts were generated for the period 2026–2030. Model-based uncertainty was reported using both 95% confidence intervals, representing uncertainty around the expected mean forecast, and 95% prediction intervals, representing uncertainty around future annual median observations. Given the limited number of annual observations, forecasts were interpreted as descriptive trend-based projections under continuation of the observed non-COVID historical pattern, rather than as deterministic predictions of future annual values. Finally, an event-exposure scenario analysis was conducted to translate forecasted Total PPE into a more practically interpretable estimate of ranking-related competitive exposure. Total PPE was used for this analysis because it was defined in relation to official total ranking points and therefore provided the most appropriate efficiency metric for estimating the number of competitive event exposures associated with selected TRP benchmarks. Estimated competitive event exposure was calculated as: estimated competitive event exposure = TRP benchmark / forecasted Total PPE. Four TRP benchmark scenarios were considered. Three benchmarks were derived from the 13 structurally defined elite players observed in 2025: the 25th percentile of TRP, the median TRP, and the 75th percentile of TRP. These represented entry-level elite, typical elite, and high elite reference scenarios, respectively. A fourth benchmark was defined as the median TRP among elite players across all non-COVID years, representing a historical elite reference scenario. For each benchmark, required competitive event exposure was calculated for each forecast year from 2026 to 2030. To propagate uncertainty in the 2025 benchmark estimates, 10,000 paired bootstrap resamples of the 2025 elite observations were generated with elite classification held fixed. Within each resample, the three TRP benchmarks and the 2025 elite median Total PPE were recalculated. The resampled 2025 median Total PPE was then substituted into the 20-observation annual Total PPE series, the linear forecasting model was refitted, and model-based uncertainty in the expected Total PPE forecast was incorporated before calculating required exposure. The 2.5th and 97.5th percentiles of the resulting exposure distributions were reported as 95% propagated uncertainty intervals. This procedure preserved the observed within-player relationship between TRP and Total PPE in the 2025 elite subset. For the historical benchmark, the TRP reference value was held fixed and only uncertainty associated with the forecasted Total PPE trajectory was propagated. These estimates were interpreted as conditional scenario-based projections rather than deterministic forecasts of the number of competitive event exposures required by individual players. Model results were reported using unstandardised regression coefficients, standard errors, t values, p values and coefficients of determination (R²). Where relevant, 95% confidence intervals and 95% prediction intervals were also reported. All analyses were conducted in R using RStudio (Posit Software, v2026.01.1, Build 403 for Windows). Data manipulation was performed using the *dplyr* and *tidyr* packages, and graphical outputs were generated using *ggplot2*.

## Results

### Analysis 1 – Performance efficiency trajectories in elite and non-elite players

A total of 8,510 player-year observations from 5,919 female junior players were analysed across the period 2004–2025. Elite status was determined using the annual knee percentile derived from the smoothed Total Singles Points profile across the TRP-based percentile ordering and subsequently applied to the same percentile scale. Overall, 907 observations were classified as elite and 7,603 observations as non-elite. To prevent the exceptionally deep 2020–2021 source-data coverage from dominating pooled descriptive estimates, Table 1 reports pooled performance-efficiency indicators after excluding these two seasons. This non-COVID descriptive sample comprised 1,869 player-year observations, including 1,562 non-elite and 307 elite observations. Full-period pooled descriptives are provided in S9 Table. Across all performance-efficiency indicators, higher pooled non-COVID PPE values were observed among structurally defined elite players than among non-elite players (Table 1). For Singles PPE, the median was 64.62 in the elite group compared with 26.56 in the non-elite group, corresponding to an elite-to-non-elite median ratio of 2.43. For Doubles PPE, the corresponding medians were 48.57 and 29.00, respectively, with a median ratio of 1.67. For Total PPE, the median was 40.38 among elite players and 17.71 among non-elite players, corresponding to a median ratio of 2.28. Thus, after removal of the exceptionally deep 2020–2021 source-data years from the pooled descriptive comparison, structurally defined elite players continued to display higher ranking-point production per competitive event exposure across singles, doubles, and total performance indicators.

**Table 1:** Pooled non-COVID performance-efficiency indicators by elite status.

PPE metric	Non-elite n	Non-elite median [IQR]	Elite n	Elite median [IQR]	Elite/non-elite median ratio	Median difference
Singles PPE	1,562	26.56 [17.50, 40.32]	307	64.62 [41.66, 111.80]	2.43	38.05
Doubles PPE	1,561	29.00 [18.75, 45.00]	307	48.57 [29.29, 89.21]	1.67	19.57
Total PPE	1,562	17.71 [12.28, 26.34]	307	40.38 [26.29, 67.83]	2.28	22.66

Pooled descriptive statistics were calculated after excluding the COVID-disrupted seasons 2020–2021. PPE = points per event; IQR = interquartile range; TRP = total ranking points. Total PPE refers to total ranking points divided by total competitive event exposure.

Mean values were consistently higher than median values, particularly in the elite group, indicating a right-skewed distribution of performance efficiency. This skewness was most evident in Singles PPE and Doubles PPE, where maximum values reached 700.00 and 750.00, respectively, among elite players. For this reason, median trajectories were treated as the primary descriptive indicator of longitudinal development, whereas mean trajectories were interpreted as supplementary evidence of upper-tail performance behaviour.

### Annual development of PPE

The annual trajectories showed that elite players consistently achieved higher PPE values than non-elite players across singles, doubles, and total performance efficiency (Fig 1). This pattern was evident throughout the observation period and became especially pronounced after the late 2010s. Selected annual comparisons illustrated greater descriptive separation between the groups in later benchmark years (Table 2). For example, the median Singles PPE difference between elite and non-elite players was 36.36 in 2004 and 120.92 in 2025. The corresponding differences were 30.24 and 94.04 for Doubles PPE and 22.48 and 82.25 for Total PPE, respectively. These comparisons are descriptive and should not be interpreted as evidence of uniform monotonic widening across the complete observation period.

**Table 2:** Selected annual median PPE differences between elite and non-elite players.

PPE metric	Year	Non-elite median	Elite median	Median difference	Median ratio
Singles PPE	2004	20.00	56.36	36.36	2.82
Singles PPE	2019	28.75	112.95	84.20	3.93
Singles PPE	2022	46.82	141.11	94.29	3.01
Singles PPE	2025	43.62	164.55	120.92	3.77
Doubles PPE	2004	20.38	50.62	30.24	2.48
Doubles PPE	2019	39.12	79.09	39.97	2.02
Doubles PPE	2022	48.71	117.60	68.89	2.41
Doubles PPE	2025	52.06	146.10	94.04	2.81
Total PPE	2004	13.06	35.54	22.48	2.72
Total PPE	2019	20.52	68.11	47.59	3.32
Total PPE	2022	30.39	91.38	60.98	3.01
Total PPE	2025	29.93	112.18	82.25	3.75

The table presents selected benchmark years to illustrate descriptive differences between structurally defined elite and non-elite players and the comparatively greater separation observed in selected later years. Years 2020–2021 were omitted because they were treated as COVID-disrupted seasons.

**Fig 1:**
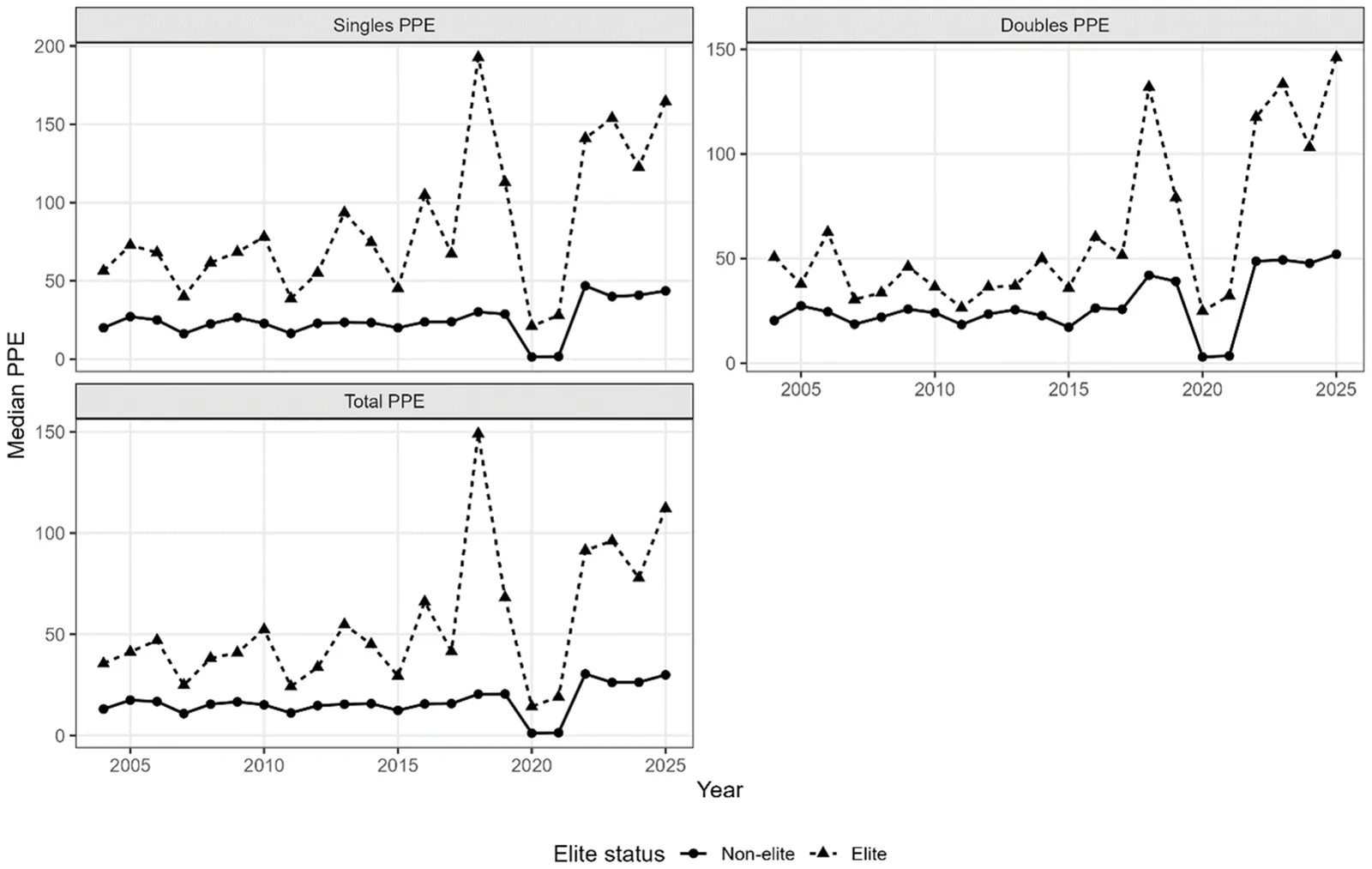
Annual median PPE trajectories by elite status among female ITF World Tennis Tour Juniors players, 2004–2025.

The COVID-disrupted years 2020–2021 produced a visible interruption in the longitudinal curves. In these years, a pronounced decline was observed across all PPE indicators, particularly in the non-elite group. Given the exceptional structure of these seasons and their unusually high number of observations, they were excluded from the linear trend models. The full trajectories including 2020–2021 were retained descriptively, whereas trend estimation was based on the non-COVID years.

### Non-COVID trend analysis

After exclusion of the COVID-disrupted years, median PPE trajectories showed a stronger upward pattern among elite players than among non-elite players across all three efficiency indicators: Singles PPE, Doubles PPE, and Total PPE (Fig 2). This visual pattern was supported by linear models fitted to annual median PPE values, in which the year-by-elite-status interaction was positive and statistically significant for all three indicators (Table 3).

**Table 3:** Linear models of annual median PPE trajectories after excluding COVID-disrupted years.

PPE metric	Non-elite annual slope, b	Elite × Year interaction, b [SE]	*t*	*p*	Elite annual slope, b	*R*²
Singles PPE	1.05	4.09 [1.06]	3.84	< .001	5.13	.792
Doubles PPE	1.48	3.26 [0.89]	3.65	< .001	4.74	.736
Total PPE	0.71	2.81 [0.85]	3.32	.002	3.52	.721

*Note*. Models were fitted to annual median PPE values after excluding 2020–2021. Year was centred at 2004. The non-elite group was used as the reference category. The Elite × Year interaction represents the additional annual change observed in elite players relative to non-elite players. The elite annual slope was calculated as the sum of the non-elite annual slope and the interaction coefficient.

**Fig 2:**
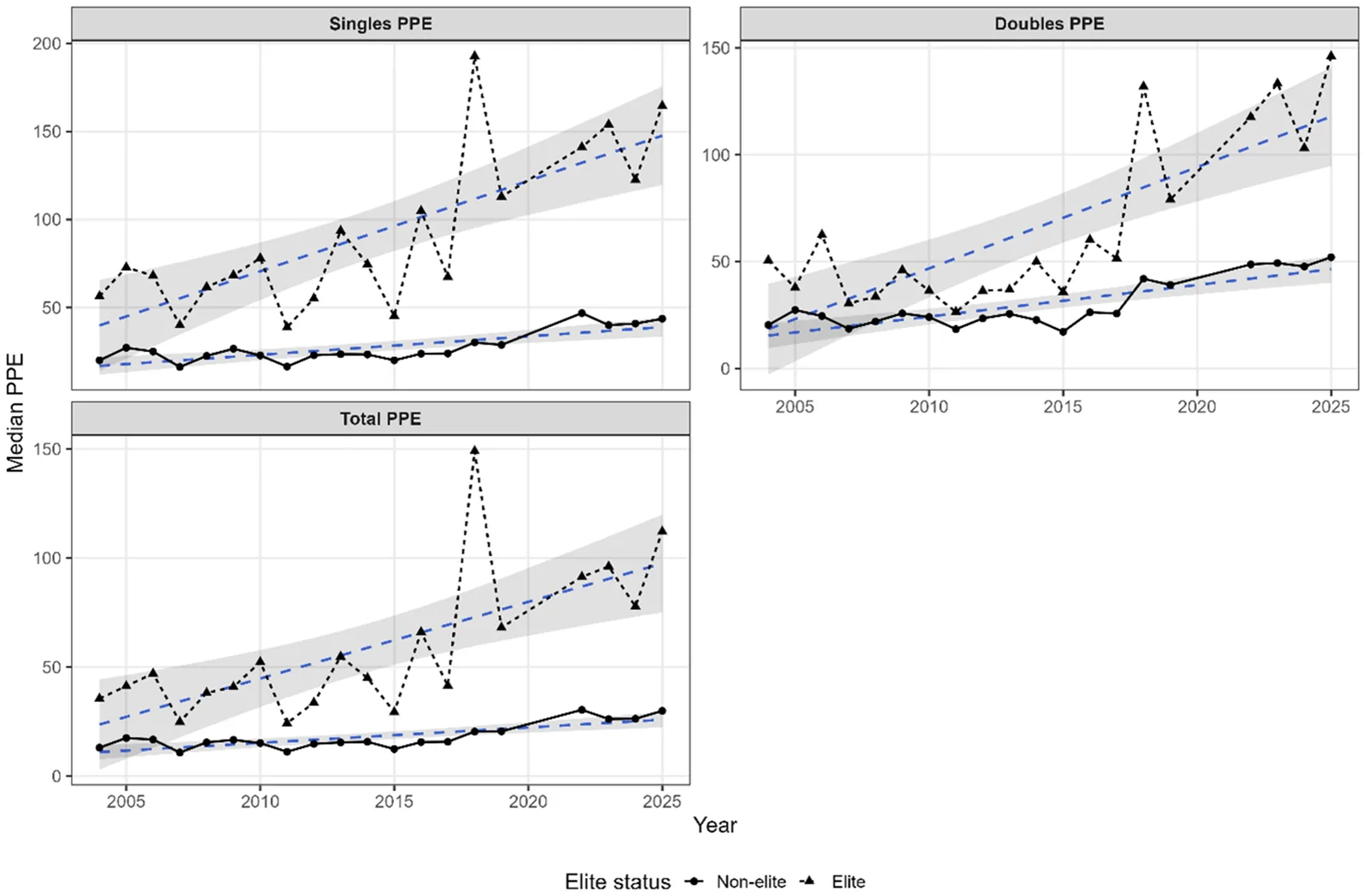
Non-COVID annual median PPE trajectories by elite status, 2004–2025.

For Singles PPE, the year-by-elite-status interaction was significant, *b* = 4.09, SE = 1.06, *t* = 3.84, *p* < .001, indicating that the annual median increase was substantially steeper among elite players than among non-elite players. The model explained a large proportion of variance in annual median Singles PPE, *R*² = .792.

For Doubles PPE, the interaction was also significant, *b* = 3.26, SE = 0.89, *t* = 3.65, *p* < .001, with the model explaining 73.6% of the variance in annual medians. Unlike Singles PPE, a significant positive annual trend was also observed in the non-elite group for Doubles PPE, *b* = 1.48, SE = 0.63, *t* = 2.34, *p* = .025. However, *t*he additional increase observed among elite players was substantially larger. For Total PPE, a significant interaction was likewise found, *b* = 2.81, SE = 0.85, *t* = 3.32, *p* = .002, wi*t*h *R*² = .721.

Taken together, the descriptive and linear model-based results indicate that elite and non-elite players differed not only in PPE level but also in their average temporal development across singles, doubles, and total PPE indicators. The positive Year × elite-status interactions in these non-COVID annual-median models indicated a steeper average linear increase among elite players, while the flexible player-year sensitivity analysis reported below showed that the overall temporal differentiation was non-linear rather than uniformly widening across the entire 2004–2025 period.

### Sensitivity analysis using year-normalised PPE

To examine whether the observed temporal divergence in performance efficiency was primarily attributable to changes in the historical ranking-point environment, a sensitivity analysis was conducted using year-normalised PPE outcomes (S2 Table). For each PPE indicator, player-year values were transformed into a within-year z-score of log1p(PPE) and a within-year PPE percentile, thereby expressing each player’s efficiency relative to the distribution observed within the same competitive year. In cluster-robust models adjusted for age, discipline-specific competitive event exposure, and COVID-disrupted years, the Year × elite-status interaction remained positive across all three PPE indicators. For the within-year z-score of log1p(PPE), the interaction was observed for Singles PPE (b = 0.060, SE = 0.007, p < .001), Doubles PPE (b = 0.053, SE = 0.009, p < .001), and Total PPE (b = 0.067, SE = 0.007, p < .001). The same pattern was reproduced using within-year PPE percentiles, with positive interactions for Singles PPE (b = 0.636, SE = 0.147, p < .001), Doubles PPE (b = 1.019, SE = 0.206, p < .001), and Total PPE (b = 0.631, SE = 0.152, p < .001). The positive elite–non-elite separation was visible for both normalisation approaches, although the magnitude of the annual gap varied across years and disciplines (S1 Fig; S2 Fig). These findings indicate that the positive linear temporal differentiation between elite and non-elite players was not solely a consequence of absolute inflation in the ranking-point scale, because a positive Year × elite-status interaction was also observed when players were compared within the same annual ranking environment.

### COVID-period sensitivity analyses

To assess whether the main trend findings were dependent on the handling of COVID-disrupted seasons, additional sensitivity analyses were performed using three alternative specifications: models including all years, models excluding 2020–2021, and models excluding 2020–2022. A retrospective data audit confirmed that the exceptional annual sample sizes were already present in the source dataset. For female players, source coverage extended to ITF rank 3,297 in 2020 and rank 2,935 in 2021, compared with rank 111 in 2019 and rank 100 in 2022. The corresponding analytical samples comprised 3,514 and 3,127 player-year observations in 2020 and 2021, respectively. No exact duplicate rows were identified, and repeated player-year records were too infrequent to explain the marked sample-size difference. The imbalance was therefore attributed to substantially deeper source-data coverage in these two seasons rather than to downstream analytical processing (S3 Table). As descriptive context for the sensitivity analyses, annual median PPE values by elite status and PPE indicator are reported in S4 Table. Across all three model specifications, the year-by-elite-status interaction remained positive for Singles PPE, Doubles PPE, and Total PPE (S5 Table). The corresponding robust regression models produced the same substantive pattern (S6 Table). These findings indicate that the positive Year × elite-status interaction in the linear annual-median models was not dependent on a single COVID-period modelling decision, although the COVID-disrupted years constituted an exceptional segment of the longitudinal dataset in terms of both competitive conditions and source-data coverage.

### Analysis 2 – Adjusted player-year models of PPE trajectories

Having established descriptive differences in the longitudinal development of ranking-derived PPE between elite and non-elite players, adjusted player-year models were fitted to examine whether these temporal differences remained evident after accounting for player age, competitive event exposure, and COVID-disrupted seasons (Table 4). Because all PPE indicators showed right-skewed distributions, the dependent variables were log1p-transformed before modelling. Separate models were fitted for Singles PPE, Doubles PPE, and Total PPE. Full model estimates, including the covariate effects of age, competitive event exposure, and COVID-disrupted years, are provided in Supplementary materials (S7 Table). The linear Year × elite-status interaction was retained as a parsimonious estimate of the average temporal difference between groups, whereas an additional natural-spline sensitivity analysis was used to determine whether the temporal trajectories remained differentiated when the linear-in-year functional-form assumption was relaxed.

**Table 4:** Year-by-elite-status interactions from adjusted player-year models of performance-efficiency trajectories.

PPE metric	*n* observations	*n* player clusters	b	Cluster-robust SE	*t*	*df*	*p*	Additional annual % change [95% CI]	*R*²
Singles PPE	8,507	5,917	0.103	0.006	16.10	5,916	< .001	10.81 [9.43, 12.20]	.668
Doubles PPE	8,382	5,830	0.086	0.007	11.86	5,829	< .001	8.97 [7.43, 10.53]	.566
Total PPE	8,510	5,919	0.088	0.006	14.89	5,918	< .001	9.25 [7.98, 10.53]	.694

The table reports the year-by-elite-status interaction from adjusted player-year models. Dependent variables were log1p-transformed. Models were adjusted for age, discipline-specific competitive event exposure, and COVID-disrupted years. Standard errors were cluster-robust and clustered at the player level. The non-elite group was used as the reference category. Additional annual percentage change was calculated as 100 × [exp(b) − 1].

Under the linear-in-year specification, the year-by-elite-status interaction was positive and statistically significant across all models, indicating a steeper average temporal increase in PPE among elite players than among non-elite players after adjustment for age, competitive event exposure, and COVID-disrupted years (Table 4). For Singles PPE, the interaction coefficient was b = 0.103, cluster-robust SE = 0.006, t = 16.10, p < .001, corresponding to an approximate additional average annual increase of 10.81% on the back-transformed log1p scale among elite players relative to non-elite players. The model explained 66.8% of the variance in log-transformed Singles PPE. A similar pattern was observed for Doubles PPE. The year-by-elite-status interaction was positive and significant, b = 0.086, cluster-robust SE = 0.007, *t* = 11.86, *p* < .001, corresponding *t*o an approximate additional annual increase of 8.97% among elite players relative to non-elite players. The model explained 56.6% of the variance in log-transformed Doubles PPE.

For Total PPE, the year-by-elite-status interaction also remained positive and significant, b = 0.088, cluster-robust SE = 0.006, *t* = 14.89, *p* < .001. This corresponded to an approximate additional annual increase of 9.25% among elite players relative to non-elite players, with the model explaining 69.4% of the variance in log-transformed Total PPE.

The flexible-year sensitivity analysis showed that the temporal trajectories of elite and non-elite players remained significantly different when year was modelled using natural cubic splines rather than as a single linear term. After exclusion of 2020–2021, joint cluster-robust Wald tests of the spline-by-elite-status interaction were significant for Singles PPE, χ²(3) = 34.79, p < .001, Doubles PPE, χ²(3) = 28.02, p < .001, and Total PPE, χ²(3) = 42.18, p < .001. The adjusted spline trajectories indicated that the between-group differentiation was non-linear, with comparatively greater separation during the later part of the observation period rather than a uniform monotonic widening across the entire 2004–2025 period (Fig 3). Consistent with this qualification, contrasts comparing the elite–non-elite difference between the 2004 and 2025 endpoints were not statistically significant in the spline models for Singles PPE (b = 0.158, 95% CI [−0.052, 0.368], p = .140), Doubles PPE (b = 0.074, 95% CI [−0.249, 0.397], p = .654), or Total PPE (b = 0.152, 95% CI [−0.044, 0.348], p = .128). Thus, the flexible specification supported significant temporal differentiation between elite and non-elite players but did not support interpreting this difference as a uniform or monotonic widening from 2004 to 2025.

**Fig 3:**
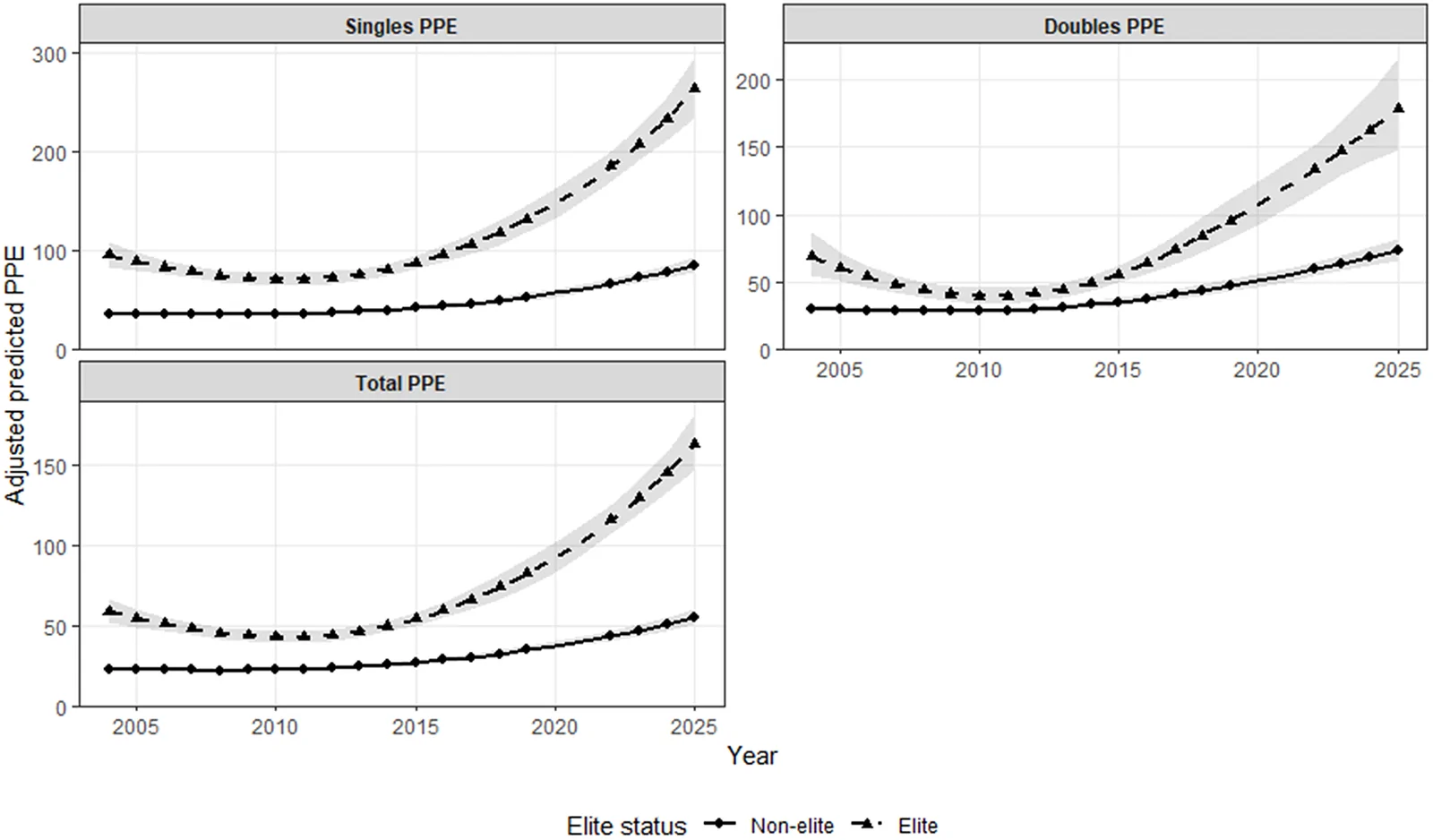
Flexible adjusted PPE trajectories by elite status after exclusion of COVID-disrupted seasons. Predicted Singles PPE, Doubles PPE, and Total PPE were obtained from player-year models in which centred year was modelled using a natural cubic spline with three degrees of freedom and interacted with elite status. The COVID-disrupted seasons 2020–2021 were excluded from model fitting. Models were adjusted for age and discipline-specific competitive event exposure, with cluster-robust standard errors at the player level. Predictions were generated at age 16 years and at median covariate values of 8 singles competitive event entries, 7 doubles competitive event entries, and 15 total competitive event exposures, respectively, and are displayed on the back-transformed log1p scale with 95% confidence intervals**.**

Age was positively associated with PPE in all linear models, indicating that higher performance efficiency was partly related to later junior age. However, temporal differences between elite and non-elite players remained evident after age and competitive event exposure were taken into account, including under the flexible non-COVID specification. COVID-disrupted years showed a large negative association with all PPE indicators in the all-years linear models, supporting the decision to treat 2020–2021 as exceptional seasons in the descriptive and sensitivity analyses. Taken together, the adjusted player-year analyses supported a temporal differentiation in ranking-point production efficiency between contemporaneously defined elite and non-elite players after age, competitive event exposure, COVID-period disruption, and repeated player observations were taken into account. However, the flexible-year sensitivity analysis qualified the interpretation of this difference. Although the temporal trajectories remained significantly different between groups, their development was non-linear and did not support a uniform monotonic widening across the complete 2004–2025 period. The positive linear Year × elite-status interaction should therefore be interpreted as a parsimonious summary of the average historical divergence rather than as evidence that the elite–non-elite difference increased at a constant rate in every part of the observation period.

### Lagged elite-status sensitivity analysis

To examine whether the difference in PPE persisted when elite-status classification was temporally separated from the outcome, an additional sensitivity analysis related elite status in the preceding year to PPE in the subsequent year (S10 Table). After construction of unique consecutive player-year pairs, the lagged dataset comprised 2,329 observations from 2,040 players. Because the all-years lagged sample remained strongly influenced by the exceptional 2020–2021 source-data coverage, primary interpretation was based on the strict non-COVID specification, which excluded outcome years 2020–2022 and comprised 583 observations from 482 player clusters.

In this strict non-COVID analysis, prior-year elite status was positively associated with PPE in the subsequent year after adjustment for year, age, and discipline-specific competitive event exposure. The association was observed for Singles PPE (b = 0.425, cluster-robust SE = 0.074, 95% CI [0.279, 0.571], p < .001), Doubles PPE (b = 0.261, SE = 0.085, 95% CI [0.094, 0.428], p = .002), and Total PPE (b = 0.385, SE = 0.069, 95% CI [0.250, 0.521], p < .001). On the back-transformed log1p scale, these coefficients corresponded to approximate differences of 52.9%, 29.9%, and 47.0%, respectively, between players classified as elite and non-elite in the preceding year.

However, the Year × prior-year elite-status interaction was not significant in the strict non-COVID specification for Singles PPE (b = −0.010, SE = 0.012, p = .426), Doubles PPE (b = −0.004, SE = 0.014, p = .782), or Total PPE (b = −0.013, SE = 0.013, p = .318). Thus, prior-year elite status was associated with higher subsequent PPE, but the magnitude of this prospective difference did not show evidence of widening over historical time. The lagged findings therefore support one-year persistence of the ranking-efficiency difference between groups, while indicating that the positive temporal interaction identified in the contemporaneous linear models should not be interpreted as evidence of an increasingly large prospective effect of prior-year elite status.

### Analysis 3 – Age profile of elite and non-elite players

As a contextual analysis, age distributions were examined to characterise developmental differences between elite and non-elite players and to contextualise the role of age in the adjusted performance-efficiency analyses. Elite players were, on average, slightly older than non-elite players. The mean age was 16.67 years among elite players and 16.25 years among non-elite players, corresponding to a mean difference of approximately 0.42 years. The median age was 17 years in the elite group and 16 years in the non-elite group. The elite group was also more concentrated in the older junior age categories, with most elite observations occurring between 16 and 18 years of age.

A simple comparison confirmed that the mean age difference between elite and non-elite players was statistically significant, p < .001. However, the magnitude of the difference was modest, and elite status alone explained only a very small proportion of age variability, R² = .010. The descriptive age profile of both groups is summarised in S8 Table. Thus, although elite players tended to be slightly older, the between-group age difference was modest relative to the substantially larger descriptive differences observed in PPE. Annual age trajectories were also examined as a developmental context (S3 Fig; S4 Fig). Across the full observation period, age patterns fluctuated between years, particularly around the COVID-disrupted seasons (S3 Fig). After excluding 2020–2021, mean age showed only a modest temporal increase in both groups (S4 Fig). In the non-COVID model, the annual age trend was positive among non-elite players, *b* = 0.028, *p* < .001, whereas the year-by-elite-status interaction was negative, *b* = −0.021, *p* = .030, indicating a slightly flatter age trajectory among elite players. The explanatory power of this model remained low, R² = .041. These findings suggest that age should be treated as a relevant contextual and adjustment variable, rather than as a primary explanation for the observed differences in performance efficiency between elite and non-elite players.

Taken together, the age-context analysis indicates that elite players were marginally older than non-elite players, but the difference was small. This supports retaining age as an adjustment variable. Importantly, ranking-efficiency differences between elite and non-elite players remained evident in the adjusted player-year analyses after age was included as a covariate.

### Analysis 4 – Forecasting elite PPE trajectories, 2026–2030

To estimate the prospective development of performance-efficiency requirements among structurally defined elite players, annual median PPE values were forecasted for the period 2026–2030. Forecasting models were fitted separately for Singles PPE, Doubles PPE, and Total PPE using 20 non-COVID annual median observations per PPE series, corresponding to 2004–2019 and 2022–2025. The COVID-disrupted years 2020–2021 were excluded from model estimation, consistent with the preceding trend analyses. The linear forecasting models indicated positive projected trajectories across all three PPE indicators (Table 5; Fig 4). The estimated annual increase was largest for Singles PPE, *b* = 5.13, *p* < .001, followed by Doubles PPE, *b* = 4.74, *p* < .001, and Total PPE, *b* = 3.52, *p* < .001. The models explained 57.3% of the variance in elite annual median Singles PPE, 62.7% of the variance in elite annual median Doubles PPE, and 49.6% of the variance in elite annual median Total PPE.

**Table 5:** Forecasted median PPE values for structurally defined elite players, 2026–2030.

PPE metric	Year	Forecasted median PPE	95% CI	95% PI
Singles PPE	2026	152.8	122.8, 182.7	83.6, 221.9
Singles PPE	2027	157.9	126.0, 189.8	87.9, 227.9
Singles PPE	2028	163.0	129.1, 196.9	92.1, 233.9
Singles PPE	2029	168.2	132.2, 204.1	96.3, 240.1
Singles PPE	2030	173.3	135.3, 211.3	100.4, 246.2
Doubles PPE	2026	122.6	97.9, 147.3	65.7, 179.6
Doubles PPE	2027	127.4	101.1, 153.7	69.7, 185.0
Doubles PPE	2028	132.1	104.1, 160.1	73.7, 190.6
Doubles PPE	2029	136.8	107.2, 166.5	77.6, 196.1
Doubles PPE	2030	141.6	110.3, 172.9	81.5, 201.7
Total PPE	2026	101.0	77.1, 125.0	45.8, 156.3
Total PPE	2027	104.5	79.0, 130.1	48.6, 160.5
Total PPE	2028	108.1	80.9, 135.2	51.4, 164.8
Total PPE	2029	111.6	82.8, 140.3	54.1, 169.1
Total PPE	2030	115.1	84.7, 145.4	56.8, 173.4

*Note.* Forecast models were fitted separately to 20 non-COVID annual median observations for each PPE series (2004–2019 and 2022–2025). Forecasted values, 95% confidence intervals (CI), and 95% prediction intervals (PI) are reported to one decimal place to reflect the limited temporal resolution of the annual series. PPE = points per event.

**Fig 4:**
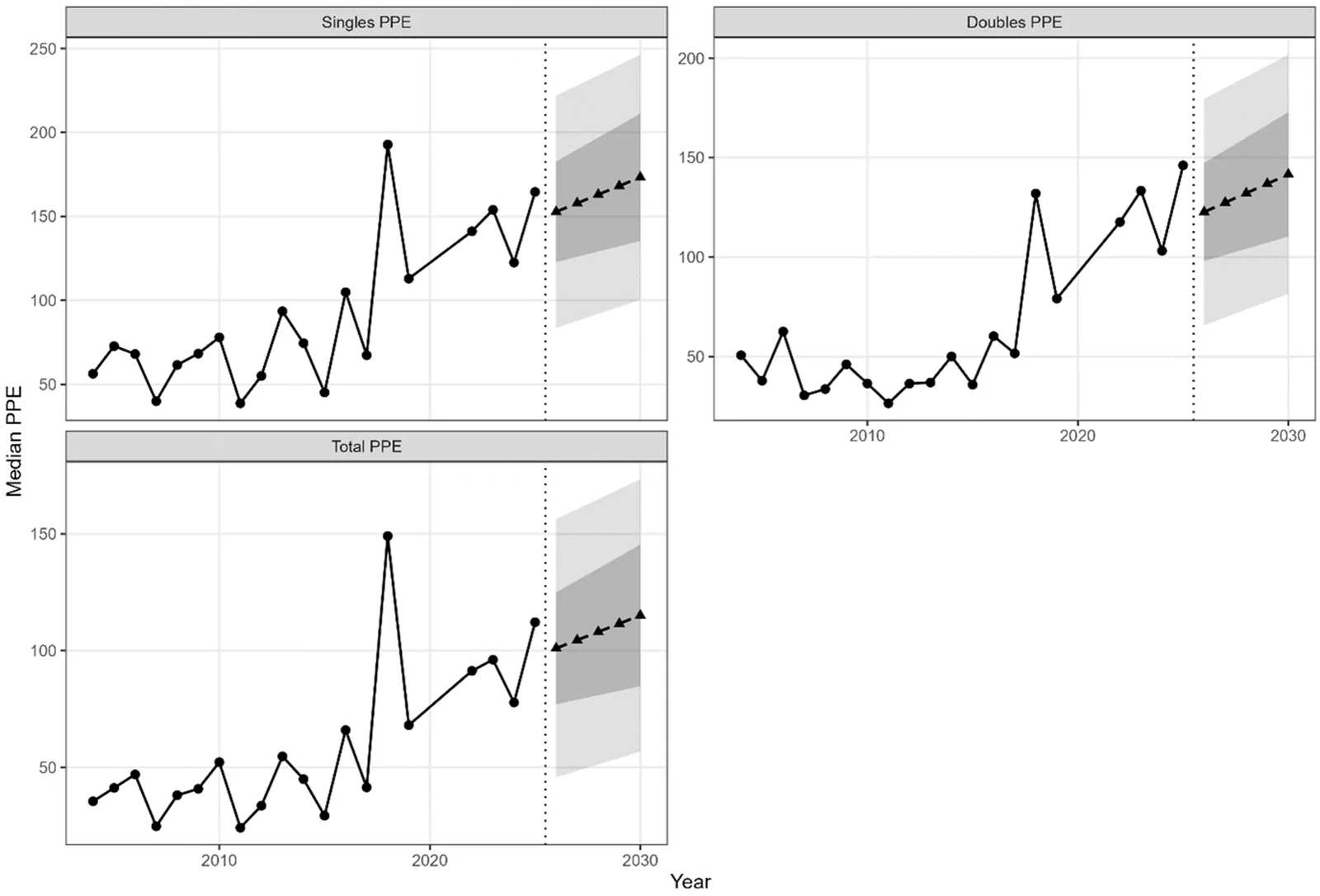
Forecasted elite median PPE trajectories, 2026–2030.

By 2030, the projected median Singles PPE among elite players was 173.3, with a 95% confidence interval of 135.3 to 211.3. The corresponding projected median value for Doubles PPE was 141.6, with a 95% confidence interval of 110.3 to 172.9. For Total PPE, the projected median value was 115.1, with a 95% confidence interval of 84.7 to 145.4. Given that each forecasting model was fitted to only 20 annual observations, these values should be interpreted as conditional continuations of the historical non-COVID trend rather than as precise estimates of future elite ranking-efficiency levels. The prediction intervals were wider than the confidence intervals, reflecting the considerable year-to-year variability in observed elite median PPE values and the limited number of annual observations available for model estimation. For example, the 2030 prediction interval ranged from 100.4 to 246.2 for Singles PPE, from 81.5 to 201.7 for Doubles PPE, and from 56.8 to 173.4 for Total PPE. Therefore, the forecasts should be interpreted as trend-based projections rather than deterministic predictions of future annual values.

Taken together, the forecasting analysis provides a conditional projection of how elite annual median PPE would develop if the observed non-COVID linear trend continued beyond the study period. The projected increase was most pronounced for Singles PPE, although positive trajectories were also obtained for Doubles PPE and Total PPE. These projections provide the empirical basis for the subsequent event-exposure scenario analysis, in which forecasted Total PPE is related to fixed TRP benchmarks to estimate corresponding competitive event exposure.

### Analysis 5 – Event-exposure scenario analysis

To translate the forecasted development of Total PPE into a more practically interpretable form, an event-exposure scenario analysis was conducted. Total PPE was used for this analysis because it was defined in relation to official total ranking points and therefore provided the most appropriate efficiency metric for estimating the number of competitive event exposures associated with selected TRP benchmarks. Required exposure was calculated as the ratio between a fixed TRP benchmark and the forecasted median Total PPE.

Four benchmark scenarios were considered: the 25th percentile, median, and 75th percentile of TRP among the 13 structurally defined elite players observed in 2025, and the median TRP among elite players across all non-COVID years. These scenarios were intended to represent entry-level elite, typical elite, high elite, and historical elite reference levels, respectively (Table 6). The corresponding 2025 TRP benchmarks were 1,981.50, 2,235.25, and 2,794.25. Bootstrap resampling confirmed appreciable finite-sample uncertainty around these values, with 95% bootstrap intervals of 1,830.50–2,407.25 for the 25th percentile, 1,981.50–2,794.25 for the median, and 2,235.25–2,896.25 for the 75th percentile. Accordingly, these values were retained as scenario-based reference points rather than interpreted as definitive normative thresholds, and their empirical uncertainty was propagated into the event-exposure estimates reported in Table 6.

**Table 6:** Event-exposure scenarios based on forecasted Total PPE in 2030.

Benchmark scenario	TRP benchmark	Forecasted Total PPE	Required exposures (95% propagated uncertainty interval)
Entry-level elite benchmark (P25 TRP in 2025)	1981.50	115.09	17.22 (13.02, 23.76)
Typical elite benchmark (median TRP in 2025)	2235.25	115.09	19.42 (14.97, 27.05)
High elite benchmark (P75 TRP in 2025)	2794.25	115.09	24.28 (17.34, 32.03)
Historical elite benchmark (median TRP across non-COVID years)	858.75	115.09	7.46 (5.56, 10.23)

*Note*. Required exposures were calculated as TRP benchmark divided by forecasted Total PPE. For the three 2025 benchmark scenarios, 95% propagated uncertainty intervals jointly incorporated empirical finite-sample uncertainty in the TRP benchmark and uncertainty in the forecasted mean Total PPE using 10,000 paired bootstrap resamples of the 2025 elite observations. Elite classification was held fixed during resampling. The historical TRP benchmark was held fixed, and its interval therefore reflects uncertainty in forecasted Total PPE rather than benchmark uncertainty. Total PPE was defined as total ranking points divided by total competitive event exposure, where total competitive event exposure represents the sum of singles and doubles event entries. Required exposures therefore refer to competitive event exposures and not necessarily to unique tournaments.

Across all benchmark scenarios, the estimated number of required competitive event exposures declined gradually from 2026 to 2030 as forecasted Total PPE increased (Fig 5). The propagated uncertainty intervals remained comparatively wide across the forecast horizon and substantially overlapped between the three 2025 benchmark scenarios, underscoring the conditional rather than deterministic nature of these projections. For the entry-level elite benchmark, defined as the 25th percentile of elite TRP in 2025, the estimated requirement decreased from 19.61 exposures in 2026 to 17.22 exposures in 2030. When rounded up to whole competitive exposures, this corresponded to a decline from 20 to 18 exposures. For the typical elite benchmark, defined as the median elite TRP in 2025, the corresponding estimate decreased from 22.13 to 19.42 exposures, equivalent to approximately 23 exposures in 2026 and 20 exposures in 2030.

**Fig 5:**
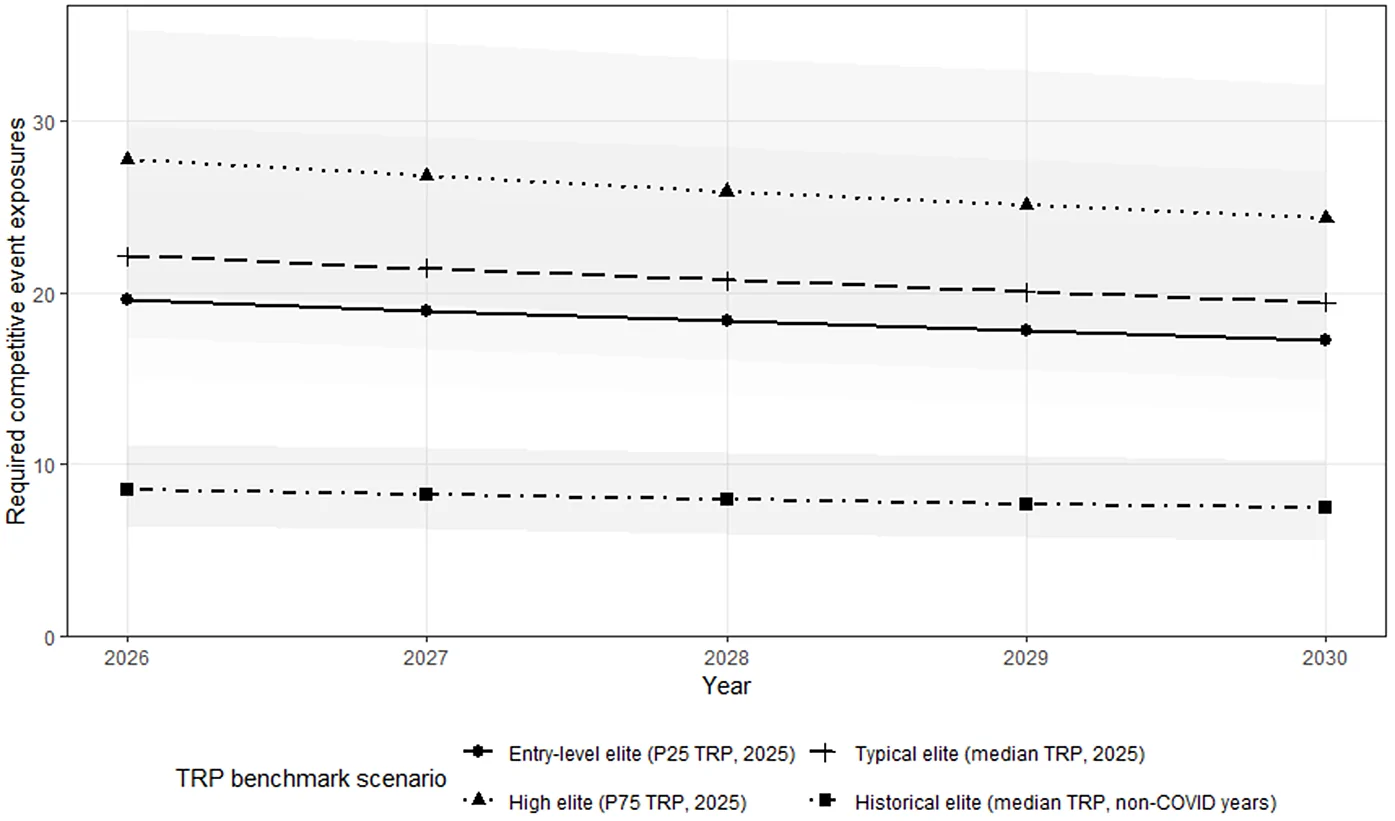
Event-exposure scenarios based on forecasted Total PPE among structurally defined elite female junior players, 2026–2030.

Lines represent point estimates of the competitive event exposure required to reach each TRP benchmark, calculated as the selected TRP benchmark divided by forecasted Total PPE. Shaded bands represent 95% propagated uncertainty intervals. For the three 2025 benchmark scenarios, these intervals jointly incorporate empirical finite-sample uncertainty in the TRP benchmark and uncertainty in forecasted mean Total PPE based on 10,000 paired bootstrap resamples. For the historical elite scenario, the TRP benchmark was held fixed and the shaded band therefore reflects uncertainty in forecasted Total PPE only. Total PPE was defined as official total ranking points divided by total competitive event exposure. The scenarios should be interpreted as conditional benchmark-based projections rather than deterministic estimates of the number of competitive event exposures required by individual players.

A higher requirement was observed for the high elite benchmark, defined as the 75th percentile of elite TRP in 2025. Under this scenario, the estimated number of required exposures declined from 27.66 in 2026 to 24.28 in 2030, corresponding to 28 and 25 rounded exposures, respectively. In contrast, the historical elite benchmark produced substantially lower estimates. Using the median TRP among elite players across non-COVID years, the required exposure decreased from 8.50 in 2026 to 7.46 in 2030, corresponding to approximately nine and eight rounded exposures, respectively. After propagation of 2025 benchmark and forecast uncertainty, the 2030 exposure estimates remained centred close to the original point estimates but were accompanied by wider uncertainty ranges: 13.02–23.76 exposures for the entry-level benchmark, 14.97–27.05 for the typical benchmark, and 17.34–32.03 for the high benchmark.

The scenario analysis should be interpreted in relation to the assumptions on which it was based. The point-value TRP benchmarks were held constant across the 2026–2030 forecast horizon, while empirical uncertainty in the three 2025 benchmark estimates was propagated into the corresponding exposure intervals. Consequently, increasing forecasted Total PPE mechanically reduced the point estimate of the number of exposures required to reach each benchmark. This does not imply that the competitive burden of elite junior tennis will necessarily decrease. Rather, it indicates that, under a fixed TRP target, higher projected point efficiency would reduce the number of competitive exposures theoretically required to reach that target. If future TRP benchmarks continue to rise, the number of required exposures may remain stable or increase despite improvements in efficiency. The analysis therefore provides a conditional scenario framework rather than a deterministic forecast of future competitive event-exposure requirements.

## Discussion

### Principal findings

The central finding of this investigation is that the structurally defined elite and non-elite segments of female junior tennis exhibited different historical trajectories in their capacity to convert competitive exposure into ranking value. Descriptive annual medians and parsimonious linear models indicated greater separation in later years, whereas the flexible-year sensitivity analysis showed that this differentiation was non-linear rather than a uniform monotonic widening across the complete 2004–2025 period. This interpretation should not be taken to imply that PPE captures sporting ability independently of the competitive and ranking context. Because PPE is derived from ranking points and competitive entries, it is best understood as an exposure-adjusted indicator of ranking-point production efficiency rather than as a direct latent measure of sporting ability. PPE values may be influenced by tournament grade, tournament selection strategy, geographical access to events, financial and federation support, singles–doubles specialisation, the number of matches won within entered events, and changes in the ranking system over time. These contextual and resource-related factors were not measured in the present dataset and therefore represent possible explanatory mechanisms rather than empirically tested determinants of the observed ranking-efficiency differences. In particular, the present study did not analyse geographic access equity, socioeconomic resources, or differences in federation support. This distinction is particularly important because successful juniors and successful seniors represent largely distinct athletic populations: previous evidence has reported only a 7.2% overlap at the international championship level, approximately 17% of junior athletes transitioning to senior national teams, and transition rates of up to 33% across sport more broadly [5,20–22]. Consequently, PPE may serve as a useful supplementary indicator for monitoring ranking-point production relative to competitive exposure. However, without direct linkage to subsequent senior outcomes, it should not be interpreted as a standalone marker of professional potential. The magnitude of ranking-efficiency differences is also context dependent, and the present analyses indicate that their historical development cannot be adequately represented by a single exponential-like back-transformed trajectory. The findings therefore support temporal differentiation in ranking-point production efficiency while requiring caution against interpreting this differentiation as a constant or uniformly accelerating historical process [3,5]. The observed increase in PPE should therefore be interpreted as a change in ranking-value production within the historical ITF junior ranking environment. Because tournament structures, point allocations, and access to higher-grade events may have changed over time, PPE cannot be treated as a direct measure of biological, technical, or tactical performance improvement independent of the ranking system.

Several non-mutually exclusive mechanisms could plausibly contribute to the observed ranking-efficiency differentiation, particularly the more pronounced separation between elite and non-elite players during the later part of the observation period. First, ranking-system mechanisms may have altered the amount of ranking value available per competitive entry through changes in point allocation, tournament-grade weighting, or the distribution of ranking-point opportunities. Second, competition-selection mechanisms may have contributed if structurally defined elite players differed in the competitive opportunities they entered, the scheduling of those opportunities, or their strategic specialisation between singles and doubles. Third, player-performance mechanisms may have contributed if elite players won more matches within entered events, progressed deeper into draws, or possessed technical, tactical, physical, or developmental characteristics that improved the conversion of competitive exposure into ranking points. Fourth, resource- and access-related mechanisms may have influenced which ranking opportunities were practically available through geographical access, financial resources, or federation support. These mechanisms may also have interacted rather than operated independently. However, the present dataset contained annual ranking-point and competitive-exposure information but did not include tournament grade, round reached, opponent quality, match-level performance, detailed scheduling strategy, financial resources, or federation support. Their relative contributions could therefore not be separated, and no causal attribution regarding the observed ranking-efficiency trajectories can be made from the present analyses.

### Performance inflation and the intensification of competition

The observed trends should be interpreted within the increasingly competitive structure of the ITF junior pathway. Since 2019, the pool of ranked junior players has reportedly expanded substantially, while the number of professional players has increased more modestly, creating a potential bottleneck between junior participation and professional entry [3,8,17]. Within such a ranking environment, cumulative ranking points alone may provide an incomplete description of competitive progression, because they are partly shaped by exposure volume and access to ranking-point opportunities. The present findings indicate persistent differences between structurally defined elite and non-elite players in their ability to produce ranking value per competitive entry, together with significant non-linear temporal differentiation between the groups. This pattern is more appropriately interpreted as ranking-efficiency differentiation than as direct evidence of biological, technical, or tactical superiority. Accordingly, the term performance-efficiency inflation is used descriptively and in a restricted ranking-system sense to denote historical changes in ranking-point production relative to competitive event exposure, including the comparatively pronounced elite–non-elite separation observed during the later part of the study period. It should not be interpreted as evidence of a constant, monotonic, or causal increase in underlying player performance [5,9,17].

### PPE as an indicator of ranking-point production efficiency

Efficiency-based indicators provide a useful complement to cumulative ranking points because they contextualise ranking outcomes in relation to competitive exposure. In the present study, PPE should be interpreted specifically as a ranking-derived indicator: Singles PPE, Doubles PPE, and Total PPE describe how efficiently players converted competitive event exposure into ranking value within the ITF junior ranking system. They should not be interpreted as direct measures of competitive maturity, professional readiness, or intrinsic sporting ability. This distinction is important because successful juniors and successful seniors constitute partly distinct athletic populations, with only limited overlap at the international level [20–22]. Traditional ranking points may reward both performance and exposure volume, whereas PPE expresses ranking-point production relative to event entries. For coaches and federations, this may provide a supplementary monitoring tool when interpreting ranking progress, particularly when cumulative ranking points are difficult to disentangle from competitive volume. However, PPE should be used alongside, rather than instead of, match-level, developmental, contextual, and longitudinal indicators, especially when making judgements about future senior potential [3,5].

### Robustness of the temporal efficiency divergence

The adjusted player-year analyses supported temporal differentiation in ranking-point production efficiency between contemporaneously defined elite and non-elite players, but the sensitivity analyses substantially refined the form in which this finding should be interpreted. Under the parsimonious linear-in-year specification, elite players showed significantly steeper average temporal increases in Singles PPE, Doubles PPE, and Total PPE after adjustment for age, competitive event exposure, COVID-disrupted seasons, and repeated player observations. However, natural-spline models fitted after exclusion of 2020–2021 demonstrated that the group trajectories were significantly different but non-linear. The spline analyses did not provide evidence of a statistically significant net increase in relative group separation between the 2004 and 2025 endpoints. The linear interaction should therefore be interpreted as an average historical contrast rather than as evidence of constant or monotonic widening throughout the full period. The lagged sensitivity analysis provided a complementary qualification: prior-year elite status remained associated with higher subsequent PPE in the strict non-COVID sample, but the Year × prior-year elite-status interactions were small and statistically non-significant across all three indicators. Taken together, these analyses support persistent ranking-efficiency differences and non-linear temporal differentiation within the annual ranking environment, but they do not support an increasingly large prospective effect of prior-year elite status or a uniform monotonic widening across the complete observation period. This interpretation remains confined to the ranking system. The present data do not identify the technical, tactical, physiological, or psychosocial mechanisms that may explain why some players convert competitive exposure into ranking points more efficiently than others. Future studies integrating ranking data with match-level performance indicators, tournament-grade information, movement characteristics, maturation status, and longitudinal senior outcomes would be required to determine the mechanisms underlying these ranking-efficiency differences [4,5,17].

### Developmental context: age and maturation

Age provides an important developmental context for interpreting ranking-point production efficiency in female junior tennis. In the present study, elite players were slightly older than non-elite players, but the between-group age difference was modest relative to the substantially larger differences observed in PPE. Age should therefore be interpreted as an important contextual and adjustment variable rather than as an empirically established mechanism underlying the observed ranking-efficiency differentiation. This interpretation is consistent with previous evidence indicating that developmental advantages associated with maturation or relative age may be important during adolescence but do not necessarily translate into sustained senior success [5]. Consistent with this broader developmental perspective, the median age for attaining elite thresholds in tennis remains lower for females than for males [23,24], with top female players typically reaching peak performance levels around age 22–24 [3,4,16]. In the present longitudinal dataset, the median age for attaining the elite threshold remained stable at approximately 16 years, even as the broader competitive base trended older. This stability suggests that the upper segment of the female junior ranking distribution may be characterised by relatively consistent age-related timing. However, because biological maturation, maturity timing, technical-tactical development, and match-level performance characteristics were not directly measured, the present study cannot determine the mechanisms underlying either this age-related stability or the observed temporal ranking-efficiency differentiation. Future research should therefore integrate chronological age with biological maturation, match-level performance indicators, tournament context, and longitudinal senior outcomes to clarify how developmental timing interacts with ranking-point production efficiency in female junior tennis.

### Forecasting and event-exposure scenario analysis

Prospective forecasting for 2026–2030 projected a continued increase in elite ranking-point production efficiency under a linear continuation of the historical non-COVID trend. These projections should be interpreted as conditional trend-based estimates rather than as deterministic predictions of future ITF ranking conditions. The forecasting component was deliberately designed as a descriptive continuation-of-trend analysis rather than as an exercise in predictive-algorithm optimisation. Because only 20 non-COVID annual median observations were available for each PPE series, estimation and validation of additional autoregressive or moving-average structure would have been inherently limited. A simple linear specification was therefore retained to maximise parsimony, transparency, and interpretability while avoiding additional model-selection uncertainty that would be difficult to resolve from such short annual series. More flexible time-series approaches may become appropriate as longer annual series accumulate or where higher-frequency observations are available. The translation of forecasted Total PPE values into event-exposure scenarios provides a conditional framework for interpreting how fixed TRP benchmarks relate mathematically to projected ranking-point production efficiency. Under these fixed-benchmark scenarios, higher projected PPE corresponds to fewer competitive event exposures associated with the same TRP target. This relationship may provide contextual information for competitive planning, but it should not be interpreted as an individual participation prescription or extended to direct conclusions about physiological workload, injury prevention, or long-term athlete health. Although the broader youth-sport literature indicates that excessive or poorly managed sport exposure may be associated with overuse injuries and burnout [25,26], the present dataset did not include match duration, number of matches played, training load, travel burden, recovery status, burnout, or injury outcomes. Therefore, any health-related interpretation of lower scenario-based event exposure remains theoretical and hypothesis-generating. Future studies combining ranking data with direct workload, recovery, and injury-surveillance measures are required to determine whether ranking-point production efficiency is associated with cumulative load or injury outcomes.

### Strengths and limitations

A major strength of this study is the use of a large longitudinal dataset covering 22 competitive seasons of the ITF World Tennis Tour Juniors, from 2004 to 2025. The analysis included 8,510 player-year observations from 5,919 female junior players, allowing long-term changes in ranking-point production efficiency to be examined across a substantial historical period. The use of year-end ranking data also provided a consistent temporal reference point for comparing annual performance patterns within the same ranking environment.

Another strength is that the study was based on publicly available ranking data. This enhances transparency, reproducibility, and practical relevance, as the analysed indicators are derived from the same ranking information used by players, coaches, federations, and tournament stakeholders. At the same time, reliance on public ranking data also constitutes an important limitation. The dataset did not include match-level information, such as number of matches played, match duration, round reached, opponent quality, scoreline characteristics, or surface-specific performance. Consequently, PPE should be interpreted as a ranking-derived efficiency indicator rather than as a direct measure of match-play quality or technical-tactical performance.

A further limitation concerns the absence of tournament-grade and access-related information. Tournament category, point allocation, draw strength, geographical access to higher-value events, and differences in financial or federation support may all influence the ranking-point opportunities available to individual players. However, these contextual and resource-related factors were not measured in the analytical dataset. The present study therefore could not distinguish efficiency differences attributable to player performance from those related to tournament selection, geographical access, resource availability, federation support, or changes in the distribution of ranking-point opportunities. Accordingly, no inference regarding geographic access equity or socioeconomic inequality can be drawn from the present analyses. This limitation is particularly relevant given potential changes in the ITF junior ranking environment across the 2004–2025 period. Ranking points in the ITF World Tennis Tour Juniors depend on tournament grade and round reached, and the structure of junior ranking points has not remained entirely static across the study period. In particular, documented reforms to the junior ranking-point structure were introduced in 2018, with greater weighting assigned to higher-grade tournaments and deeper tournament progression. Because tournament-grade information was not available in the present player-year dataset, grade-adjusted PPE could not be calculated. Consequently, the observed increase in PPE should be interpreted as an increase in ranking-value production efficiency within the historical ITF junior ranking system, rather than as direct evidence of biological, technical, or tactical performance improvement independent of ranking-rule changes.

The study also did not include data on training load, match load, travel burden, injury occurrence, burnout, or recovery status. Therefore, the event-exposure scenarios should not be interpreted as direct measures of physiological load or health risk. They provide a conditional ranking-exposure framework showing how fixed TRP benchmarks relate to projected Total PPE values, but they do not allow conclusions about safe workload thresholds, injury prevention, or long-term athlete health.

Similarly, biological maturation, maturity timing, and relative age were not directly measured. Chronological age was included as a developmental covariate, but it cannot fully capture inter-individual differences in maturation, physical development, psychosocial readiness, or training history. This is particularly relevant in female junior tennis, where developmental differences during adolescence may influence both competitive access and ranking outcomes.

It should also be noted that the elite-status variable represented an operational, ranking-based classification rather than an externally validated marker of junior talent, biological maturity, or subsequent professional success. Accordingly, the elite/non-elite distinction should be interpreted as a structural classification within the ITF junior ranking system, not as an independent latent performance construct.

A further limitation concerns both the mathematical relationship between elite-status classification and contemporaneous PPE outcomes and the functional form used to summarise their temporal development. Elite status was defined using a knee percentile derived from the Total Singles Points profile across a TRP-based percentile ordering and subsequently applied to that same TRP-based percentile scale, whereas the PPE indicators also included ranking points in their numerator. Consequently, contemporaneous classification and outcome variables were not fully independent. The lagged sensitivity analysis partially addressed this dependence by relating elite status in the preceding year to PPE in the subsequent year. Prior-year elite status remained positively associated with subsequent Singles PPE, Doubles PPE, and Total PPE in the strict non-COVID models, indicating that the observed group difference was not solely a consequence of contemporaneously using ranking-point information in both classification and outcome. However, the Year × prior-year elite-status interactions were small and statistically non-significant across all three PPE indicators. A separate natural-spline sensitivity analysis further showed that the temporal differences between contemporaneously defined elite and non-elite players were significantly non-linear. Although the spline-by-elite-status interactions remained significant, contrasts between the 2004 and 2025 endpoints did not provide evidence of a statistically significant net widening across the complete period. Accordingly, the positive interactions from the linear-in-year models should be interpreted as parsimonious summaries of average historical divergence rather than as evidence of constant exponential growth or uniform monotonic widening. The principal longitudinal finding is therefore one of ranking-derived group differentiation and non-linear temporal change within the ITF junior ranking environment, rather than an increasingly large prospective effect of prior-year elite status or an independent latent performance process. PPE should likewise not be interpreted as an independent measure of intrinsic ability or future professional potential. The forecasting analysis should also be interpreted cautiously. Forecasts were based on only 20 non-COVID annual median observations for each PPE series, which limited the temporal information available for model estimation and discouraged more elaborate time-series parameterisation. The projected values for 2026–2030 therefore represent conditional trend-based estimates under continuation of the observed non-COVID historical pattern, rather than deterministic predictions of future ranking requirements. In addition, the 2025 TRP benchmarks used in the scenario analysis were derived from only 13 structurally defined elite observations and may reflect year-specific characteristics of the most recent season. Bootstrap resampling was used to quantify and propagate empirical finite-sample uncertainty in these benchmarks into the event-exposure scenarios. However, this procedure was conditional on the observed 2025 elite classification and ranking distribution and should not be interpreted as converting these empirical reference values into population-level normative standards. Finally, the study was restricted to female junior players. This design choice improved conceptual homogeneity and avoided conflating sex-specific developmental and competitive dynamics. However, the findings cannot be directly generalised to male junior players or to other developmental pathways without further empirical validation. Future research should examine whether comparable ranking-efficiency trajectories are observed in boys’ junior tennis, whether PPE indicators predict subsequent senior outcomes, and whether ranking-derived efficiency measures can be integrated with match-level, tournament-grade, maturation, and workload data.

## Conclusion

This study demonstrates that ranking-point production efficiency in female junior tennis has changed substantially over the past two decades. Structurally defined elite players displayed higher points-per-event values than non-elite players, and the two groups followed significantly different historical trajectories across singles, doubles, and total PPE indicators. Descriptive and linear trend analyses indicated greater separation in later years, whereas flexible-year modelling showed that this temporal differentiation was non-linear and should not be interpreted as a uniform monotonic widening across the entire 2004–2025 period. The use of an annual knee percentile derived from the Total Singles Points profile across the TRP-based percentile ordering allowed elite status to be defined from the internal structure of the ranking distribution rather than from an arbitrary fixed threshold. The resulting knee percentile was applied to the same TRP-based percentile scale to distinguish the structurally defined upper segment of the junior ranking system and to examine how performance efficiency evolved within and beyond this group. Under parsimonious linear specifications, elite players displayed steeper average temporal increases in Singles PPE, Doubles PPE, and Total PPE than non-elite players after adjustment for age, competitive event exposure, COVID-disrupted seasons, and repeated player observations. Flexible natural-spline models confirmed significant temporal differences between the groups but showed that these trajectories were non-linear and did not provide evidence of a statistically significant net widening between the 2004 and 2025 endpoints. A lagged sensitivity analysis further showed that prior-year elite status was associated with higher subsequent PPE across all three indicators in a strict non-COVID sample, whereas Year × prior-year elite-status interactions were not statistically significant. The longitudinal findings should therefore be interpreted as evidence of ranking-derived group differences and non-linear temporal differentiation within the ITF junior ranking environment, rather than as evidence of an independent latent performance trait, uniform monotonic widening, or a progressively increasing prospective effect of elite status. The contextual age analysis showed that elite players were slightly older than non-elite players, but the magnitude of this difference was modest. Age and competitive event exposure should therefore be interpreted as relevant contextual and adjustment variables. Importantly, the temporal differences between elite and non-elite players remained evident in the adjusted models after both variables were included, although the observational design does not establish the causal contribution of either factor. The forecasting analysis extended these findings prospectively. Under continuation of the non-COVID historical trend, elite median PPE values were projected to increase further between 2026 and 2030, particularly in singles performance. The event-exposure scenario analysis translated these projected Total PPE values into conditional benchmark-based estimates of ranking-related competitive exposure. These scenarios should not be interpreted as deterministic predictions of the number of competitive event exposures required by individual players, nor as indicators of physiological load, injury risk, or safe workload thresholds. Rather, they provide a conditional framework showing how fixed TRP targets relate to projected efficiency levels.

Overall, the study indicates that cumulative ranking points and exposure-adjusted ranking efficiency provide complementary information about the ITF junior ranking environment. Points-per-event indicators offer coaches, federations, and researchers an additional descriptive perspective for contextualising ranking-point accumulation in relation to competitive event exposure. PPE may therefore be useful for ranking monitoring and longitudinal analysis of system-level competitive patterns, but it should not be used in isolation for talent identification, developmental evaluation, or prediction of subsequent professional success. In conclusion, the structurally defined elite segment of female junior tennis was consistently characterised by greater ranking-point production efficiency than the non-elite segment, and the historical development of this difference was not constant across the 2004–2025 period. The evidence supports significant, non-linear temporal differentiation in the conversion of competitive exposure into ranking value, with comparatively pronounced separation during the later part of the observation period. This ranking-efficiency differentiation represents a meaningful feature of the evolving ITF junior ranking environment, while remaining a ranking-derived pattern that should not be interpreted as a standalone measure of intrinsic sporting potential.

## Supporting information

S1 Table Annual knee-point thresholds used for elite-status classification among female ITF World Tennis Tour Juniors players, 2004–2025. Within each year, players were ordered according to Total Ranking Points (TRP), with lower percentile values representing stronger ranking positions. The annual knee percentile was derived from the smoothed profile of Total Singles Points across this TRP-based percentile ordering and was subsequently applied to the same percentile scale. Players whose TRP-based percentile position was less than or equal to the annual knee percentile were classified as elite, whereas all remaining players were classified as non-elite. The column “n classification mismatches” verifies the internal consistency between the stored elite-status classification and this annual knee-percentile rule; no classification mismatches were identified. No new elite-status classification was created for this supplementary table.
(XLSX)

S2 Table Year-normalised PPE sensitivity analyses using within-year z-scores and percentiles. PPE = points per event; TRP = total ranking points; CI = confidence interval. The table reports Year × elite-status interaction estimates from cluster-robust player-year models using year-normalised PPE outcomes. Two normalisation approaches were used for each PPE indicator: within-year z-scores of log1p(PPE) and within-year PPE percentiles. These transformations express each player’s ranking-point production efficiency relative to the distribution observed within the same competitive year. Models were adjusted for age, discipline-specific competitive event exposure, and COVID-disrupted years, with standard errors clustered at the player level. The non-elite group was used as the reference category. The coefficient b represents the additional annual change in the year-normalised PPE outcome among elite players relative to non-elite players. Positive coefficients indicate a positive linear temporal differentiation between elite and non-elite players after normalising PPE within each competitive year; they should not be interpreted as evidence of uniform monotonic widening across the complete observation period.
(XLSX)

S3 Table Annual source-data coverage and analytical player-year observation counts by elite status and COVID-period classification. The table reports the number of female records represented in the original source dataset, the deepest observed ITF ranking position within each year, and the corresponding number of player-year observations retained in the analytical dataset. Analytical observations are additionally reported by elite status and according to the availability of Singles PPE, Doubles PPE, and Total PPE. The COVID-period classification identifies the COVID-disrupted seasons and the immediate post-COVID transition year used in the sensitivity analyses.
(XLSX)

S4 Table Annual median PPE values by elite status and PPE indicator. The table presents annual median values for Singles PPE, Doubles PPE, and Total PPE separately for elite and non-elite players, together with the corresponding number of valid player-year observations. Elite status was defined using the annual knee-point classification procedure described in the Methods.
(XLSX)

S5 Table COVID-period sensitivity analysis of linear annual median PPE trend models. The table reports linear regression models estimating annual median PPE trends under three COVID-period specifications: all years included, exclusion of 2020–2021, and exclusion of 2020–2022. The non-elite annual slope represents the estimated yearly change among non-elite players, whereas the Elite × Year interaction represents the additional yearly change among elite players. A positive interaction indicates a steeper average linear annual change among elite players relative to non-elite players under the specified model; it should not be interpreted as evidence of uniform monotonic widening across the complete observation period.
(XLSX)

S6 Table Robust regression sensitivity analysis of annual median PPE trends under alternative COVID-period specifications. The table reports robust regression models fitted as a complementary sensitivity check against potentially influential annual values. The model specifications correspond to those in S5 Table. The non-elite annual slope, Elite × Year interaction, and elite annual slope have the same interpretation as in the linear models; robust-regression p-values should be interpreted as approximate sensitivity evidence rather than as primary inferential estimates.
(XLSX)

S7 Table Full adjusted player-year models with cluster-robust standard errors. The table reports the full estimates from adjusted player-year models fitted separately for Singles PPE, Doubles PPE, and Total PPE. Dependent variables were log1p-transformed before modelling. Year was centred at 2004. Elite status was entered as a categorical predictor, with non-elite players used as the reference group. The main effect of Year therefore represents the annual change among non-elite players, whereas the Year × elite-status interaction represents the additional annual change among elite players relative to non-elite players. Models were adjusted for age, discipline-specific competitive event exposure, and COVID-disrupted years. Competitive event exposure was represented by the number of singles competitive event entries in the Singles PPE model, the number of doubles competitive event entries in the Doubles PPE model, and total competitive event exposure in the Total PPE model. COVID-disrupted years were coded as a binary covariate for the 2020 and 2021 seasons. Standard errors were cluster-robust and clustered at the player level to account for repeated player-year observations. Percent change and corresponding confidence intervals were calculated from the log-scale coefficients as 100 × [exp(b) − 1]. PPE = points per event; SE = standard error; CI = confidence interval; R² = coefficient of determination; adj. R² = adjusted coefficient of determination.
(XLSX)

S8 Table Age profile of elite and non-elite female junior players. The table summarises age distributions by elite-status group using player-year observations. Elite status was defined annually using the knee-point classification procedure. Age is reported in years. n observations refers to the number of player-year observations, whereas n players refers to the number of unique players represented within each group. Mean values are reported with standard deviations, and medians are reported with interquartile ranges. Because players could contribute observations across multiple competitive years, the number of observations exceeds the number of unique players.
(XLSX)

S9 Table Full-period pooled performance-efficiency indicators by elite status, 2004–2025. The table reports pooled descriptive statistics for Singles PPE, Doubles PPE, and Total PPE across the complete analytical dataset, including the COVID-disrupted seasons 2020–2021. Because these seasons were characterised by exceptionally deep source-data coverage, the full-period pooled estimates are provided as supplementary descriptive context, whereas the primary pooled descriptives excluding 2020–2021 are reported in Table 1.
(XLSX)

S10 Table Lagged elite-status sensitivity analyses of subsequent performance efficiency. The table reports sensitivity analyses relating elite status in the preceding competitive year to Singles PPE, Doubles PPE, and Total PPE in the subsequent year. Year × prior-year elite-status interactions are presented under three COVID-period specifications, together with strict non-COVID models estimating the overall association between prior-year elite status and subsequent PPE. The analysis was used to evaluate whether the principal findings persisted when elite-status classification and PPE outcome were temporally separated.
(XLSX)

S1 Fig Elite-non-elite gap in year-normalised PPE percentile. (TIF)

S2 Fig Elite-non-elite gap in year-normalised PPE; Mean within-year z-score of log1p(PPE). (TIF)

S3 Fig Annual mean age trajectories by elite status. The figure shows annual mean age trajectories for structurally defined elite and non-elite female players on the ITF World Tennis Tour Juniors across the full observation period. Points represent annual mean age values based on player-year observations. Linear trend lines were fitted separately by elite status, with shaded bands indicating 95% confidence intervals. The figure provides developmental context for interpreting the adjusted PPE models and illustrates year-to-year fluctuations in mean age, including the COVID-disrupted seasons.
(TIF)

S4 Fig Non-COVID annual mean age trajectories by elite status. The figure shows annual mean age trajectories for elite and non-elite female players after excluding the COVID-disrupted seasons 2020–2021. Points represent annual mean age values based on player-year observations. Linear trend lines were fitted separately by elite status, with shaded bands indicating 95% confidence intervals. This sensitivity visualisation was used to assess whether age trajectories were materially affected by the COVID-disrupted period. The observed temporal pattern indicates only a modest increase in mean age over time, supporting the interpretation of age as a contextual covariate rather than as an empirically established mechanism underlying the observed ranking-efficiency differentiation.
(TIF)

S1 File Anonymised analysis-ready dataset. The file contains the player-year data underlying the reported analyses, including stable anonymised player identifiers, ranking and exposure variables, PPE indicators, TRP-based percentile positions, annual knee percentiles, and structural elite-status classifications. An audit sheet and data dictionary are included within the workbook.
(XLSX)

S2 File R analysis code. Author-generated R code used to reproduce the data processing, descriptive analyses, adjusted player-year models, year-normalised sensitivity analyses, flexible-year spline models, lagged elite-status sensitivity analysis, forecasting analyses, bootstrap uncertainty propagation, event-exposure scenarios, and graphical outputs reported in the manuscript.
(TXT)
