# Supplementary material for: Rising performance-efficiency demands in female junior tennis: A longitudinal analysis of ITF World Tennis Tour Juniors rankings, 2004–2025
Source: PLoS One. 2026 Sep 29;21(9):e0359538. doi: 10.1371/journal.pone.0359538 (PMC13623148; doi:10.1371/journal.pone.0359538)
Supplement: Supplementary file 1 [file pone.0359538.s018.docx]

Journal RequirementPONE-D-26-25219

Rising performance-efficiency demands in female junior tennis: A longitudinal analysis of ITF World Tennis Tour Juniors rankings, 2004–2025

PLOS One

# Journal Requirements

When submitting your revision, we need you to address these additional requirements.

1.Please ensure that your manuscript meets PLOS ONE's style requirements, including those for file naming. The PLOS ONE style templates can be found at

<https://journals.plos.org/plosone/s/file?id=wjVg/PLOSOne_formatting_sample_main_body.pdf> and

[https://journals.plos.org/plosone/s/file?id=ba62/PLOSOne_formatting_sample_title_authors_affiliations.pdf](https://track.editorialmanager.com/CL0/https:%2F%2Fjournals.plos.org%2Fplosone%2Fs%2Ffile%3Fid=ba62%2FPLOSOne_formatting_sample_title_authors_affiliations.pdf/1/010f019f5a09eace-6a2ce74f-4200-476f-a502-3621e6a86ee2-000000/9OlMkUKSyM5z8i8XFHaDghKJ45RVyrUlVOjCMN8Zwao=258)

**Response:** The revised manuscript and accompanying files have been reviewed and formatted in accordance with the PLOS ONE style and file-naming requirements.

2. Please note that PLOS One has specific guidelines on code sharing for submissions in which author-generated code underpins the findings in the manuscript. In these cases, we expect all author-generated code to be made available without restrictions upon publication of the work. Please review our guidelines at <https://journals.plos.org/plosone/s/materials-and-software-sharing#loc-sharing-code> and ensure that your code is shared in a way that follows best practice and facilitates reproducibility and reuse.

**Response:** The author-generated analysis code underlying the findings has now been prepared and is provided as S2 File (R analysis code). The script reproduces the principal data-processing and analytical workflow from the anonymised analysis-ready dataset supplied as S1 File, including PPE derivation, annual elite-status classification checks, pooled and annual descriptive analyses, COVID-period sensitivity analyses, cluster-robust player-year models, year-normalised PPE analyses, flexible-year natural-spline sensitivity models, the lagged elite-status sensitivity analysis, age-context analyses, linear forecasting, bootstrap propagation of benchmark uncertainty, event-exposure scenarios, and the corresponding graphical outputs. A fixed random seed is specified for bootstrap reproducibility, required R packages are declared within the script, and sessionInfo() is exported as part of the reproducibility output. The manuscript and Supporting Information list have been revised to identify S1 File as the anonymised analysis-ready dataset and S2 File as the complete author-generated R analysis code. During construction of the reproducibility dataset, the stable anonymised player identifier also confirmed that the 8,510 analytical records represented 5,919 unique players; the previously reported value of 5,916 was therefore corrected throughout the manuscript.

3. If the reviewer comments include a recommendation to cite specific previously published works, please review and evaluate these publications to determine whether they are relevant and should be cited. There is no requirement to cite these works unless the editor has indicated otherwise.

**Response:** The publications specifically raised during peer review were evaluated for relevance. The two closely related 2026 publications were retained and are now discussed explicitly in the Introduction, where their shared data provenance and the distinct analytical contribution of the present study are delineated.

# Reviewer #1:

This manuscript examines longitudinal changes in ranking-point production efficiency among female ITF World Tennis Tour Juniors players from 2004–2025, using a data-driven knee-point procedure to define elite status and adjusted player-year models with cluster-robust standard errors. The topic is relevant to talent development research, the dataset is large, the author is commendably transparent about the limitations of ranking-derived metrics, and the raw data are provided. The sensitivity analyses strengthen the work. However, several issues should be addressed before the manuscript is suitable for publication.

Inconsistency in the elite-classification variable. The Abstract and Methods (lines 286–290) state that the knee-point procedure was applied to the singles ranking-point distribution, whereas the Results (lines 445–446) and Conclusion (line 1000) state that elite status was based on total ranking points (TRP). These cannot both be correct. Please state unambiguously which distribution was used, and ensure the description is consistent throughout the Abstract, Methods, Results, and S1 Table caption.

**Response:** The apparent inconsistency was investigated by reconstructing the elite-classification workflow from the analytical dataset. The stored percentile variable was confirmed to represent annual TRP-based ordering, whereas the annual knee percentile was derived from the smoothed profile of Total Singles Points across this ordering. The resulting knee percentile was subsequently applied to the same TRP-based percentile scale, with players classified as elite when their percentile position was less than or equal to the annual knee percentile. This rule reproduced the stored elite classification exactly across all 8,510 observations and in every year from 2004 to 2025, with zero classification mismatches. The classification procedure has therefore been described explicitly as a two-stage process and the terminology has been made consistent throughout the Abstract, Methods, Results, Conclusion, and S1 Table caption.

Extreme imbalance in annual sample sizes. According to S3 Table, the 2020 and 2021 seasons contain approximately 3,514 and 3,127 player-year observations respectively — together roughly 78% of the entire dataset — while the remaining 20 seasons appear to average fewer than 100 observations per year. This requires explicit explanation: why do the year-end ranking archives differ so dramatically in depth across years? Is this a property of the ITF ranking lists themselves or of data collection? This imbalance has consequences beyond the COVID exclusion: (a) the overall descriptive statistics in Table 1 (e.g., non-elite median Singles PPE = 2.50) are dominated by the COVID-disrupted seasons and are inconsistent with the annual medians shown in Figure 1 and Table 2 (non-elite annual medians of roughly 20–50), which may mislead readers; (b) knee-point estimation and within-year percentiles are computed on cohorts of very different sizes, which may affect classification stability. I recommend reporting Table 1 descriptives with COVID years excluded (or both versions), and discussing how list depth differences affect the knee-point procedure.

**Response:** The marked imbalance in annual sample sizes was investigated through a retrospective audit of the original source dataset and the subsequent analytical workflow. The unusually large 2020–2021 samples were confirmed to be present before the construction of the analytical dataset and were therefore not introduced by subsequent modelling or filtering. Among female players, the source dataset contained 111 records and extended to ITF rank 111 in 2019, whereas coverage increased to 3,514 records extending to rank 3,297 in 2020 and 3,137 records extending to rank 2,935 in 2021, before returning to 100 records extending to rank 100 in 2022. No exact duplicate rows were identified. Repeated player-year keys were observed in 2020 and 2021, but their frequency was insufficient to explain the marked increase in annual sample size. The imbalance was therefore attributable primarily to substantially deeper ranking coverage in the source data for the COVID-disrupted seasons. Because row-level source metadata from the historical extraction were not retained, it cannot be determined retrospectively whether the difference originated from the depth of the ITF archive display itself or from the depth of the original data extraction; this uncertainty has now been stated explicitly in the Methods. The description of the 2020–2021 seasons has also been revised in the Methods and Results to distinguish source-data coverage from downstream analytical processing, and S3 Table has been expanded to report annual female source-record counts, the deepest observed ITF ranking position, analytical sample sizes, and COVID-period classification.

To address the reviewer’s concern that the pooled descriptive statistics in the original Table 1 were dominated by the exceptionally large 2020–2021 samples, the primary pooled descriptive analysis was recalculated after excluding these two seasons. This retained 1,869 player-year observations from the 20 non-COVID seasons. The effect on the pooled estimates was substantial: the non-elite median Singles PPE increased from 2.50 to 26.56, Doubles PPE from 4.83 to 29.00, and Total PPE from 1.97 to 17.71. The corresponding elite medians increased from 34.91 to 64.62, from 33.60 to 48.57, and from 22.66 to 40.38, respectively. Consequently, the elite-to-non-elite median ratios were revised from 13.96 to 2.43 for Singles PPE, from 6.95 to 1.67 for Doubles PPE, and from 11.52 to 2.28 for Total PPE. The revised non-COVID pooled estimates are now reported as Table 1 and are substantially more consistent with the year-specific non-COVID medians shown in the longitudinal analyses. For transparency, the original full-period pooled descriptives, including 2020–2021, have been retained in S9 Table. The corresponding values in the Abstract and Results have been updated accordingly.

The reviewer’s additional concern regarding the effect of unequal annual list depth on the knee-point procedure was also considered explicitly. Because the elite boundary was estimated separately within each year on a TRP-based percentile scale, it was not determined by a fixed absolute ranking position or a fixed number of players. Nevertheless, substantially deeper ranking coverage can affect both the resolution of the annual percentile scale and the empirical shape of the smoothed Total Singles Points profile from which the knee is identified. The 2020–2021 knee estimates should therefore not be assumed to be directly comparable, in a measurement sense, with estimates derived from substantially shallower annual lists.

The annual classification audit nevertheless confirmed that the implemented classification rule was internally consistent in every season, with zero mismatches between the stored elite-status classification and the reconstructed annual knee-percentile rule. In 2020 and 2021, the estimated knee percentile was 9% in both seasons, classifying 317 of 3,514 observations (9.02%) and 283 of 3,127 observations (9.05%) as elite, respectively. For contextual comparison, the knee percentiles were 10% in 2019 and 11% in 2022. S1 Table now reports the annual sample size, knee percentile, elite and non-elite counts, percentile boundaries, ranking-point boundaries, and classification-consistency checks for every season. These checks demonstrate internal reproducibility of the applied classification but are not interpreted as evidence that the knee estimate is invariant to annual list depth.

Because the exceptional 2020–2021 list depth remains a potential source of structural non-comparability, these seasons were excluded from the primary annual trend models and were retained only in the full descriptive trajectories; alternative COVID-period exclusions were additionally examined in sensitivity analyses. A post hoc truncation of the 2020–2021 lists to an arbitrarily selected common ranking depth was not introduced, because such truncation would itself impose a new fixed selection rule and modify the empirical distribution from which the structural knee was estimated. The revised manuscript therefore acknowledges annual list depth as a limitation of cross-year comparability of the knee threshold rather than assuming that the knee-point procedure is unaffected by cohort size.

Circularity between classification and outcome. The author acknowledges that elite status is derived from ranking points while PPE contains ranking points in its numerator, and the interpretation is appropriately restricted. However, given this dependence, I would encourage the author to consider (or at least discuss) a classification based on the prior year's ranking, or on event counts plus a points threshold lagged in time, as a robustness check. At minimum, the limitation should be stated earlier (Introduction/Methods) rather than primarily in the Discussion.

**Response**: To address the reviewer’s concern regarding the contemporaneous mathematical dependence between elite-status classification and PPE, an additional lagged sensitivity analysis was conducted in which elite status in year t−1 was related to PPE in year t among players represented in consecutive seasons. Repeated player-year keys in the lagged sample were audited before modelling: model-equivalent duplicate records were collapsed to a single observation, whereas one duplicated player-year key containing discrepant PPE and exposure values was excluded rather than resolved arbitrarily. The resulting lagged dataset comprised 2,329 observations from 2,040 players. Because the lagged all-years sample remained strongly influenced by the exceptionally deep 2020–2021 source-data coverage, three specifications were examined: all available consecutive years, exclusion of outcome years 2020–2021, and a strict non-COVID specification excluding outcome years 2020–2022 so that neither the outcome nor its immediately preceding elite-status classification originated from a COVID-disrupted season.

In the strict non-COVID sample, which comprised 583 observations from 482 player clusters, prior-year elite status remained positively associated with subsequent PPE after adjustment for year, age, and discipline-specific competitive event exposure. The association was observed for Singles PPE (b = 0.425, cluster-robust SE = 0.074, 95% CI [0.279, 0.571], p < .001), Doubles PPE (b = 0.261, SE = 0.085, 95% CI [0.094, 0.428], p = .002), and Total PPE (b = 0.377, SE = 0.071, 95% CI [0.237, 0.516], p < .001). These results indicate that players classified as elite in the preceding year continued to show higher ranking-point production efficiency in the subsequent year, even when classification and outcome were temporally separated.

The lagged analysis nevertheless provided an important qualification to the primary longitudinal finding. Although the Year × prior-year elite-status interactions were positive in the all-years specification, this specification was strongly influenced by the exceptional 2020–2021 source-data coverage. In the strict non-COVID specification, the interactions were small and statistically non-significant for Singles PPE (b = −0.011, p = .415), Doubles PPE (b = −0.004, p = .782), and Total PPE (b = −0.013, p = .318). Thus, the lagged analysis supported an overall prospective association between prior-year elite status and higher subsequent PPE, but did not reproduce a positive temporal interaction over historical time. The manuscript has therefore been revised to distinguish more clearly between the positive average temporal interaction observed in the contemporaneous linear models and the absence of evidence for an increasingly large prospective difference in the lagged analysis. This sensitivity analysis does not eliminate the ranking-derived nature of both constructs, but it removes their contemporaneous mathematical dependence. Full results are reported in S10 Table, and the ranking-derived dependence between contemporaneous elite-status classification and PPE has now been stated explicitly in the Introduction as well as addressed in the Methods, Results, and limitations.

Adjusted prediction curves (Figure 3). The log-linear specification implies exponential growth on the back-transformed scale, and the adjusted predicted elite Singles PPE by 2025 (~390) is far above the observed 2025 elite median (~165). Please state at which covariate values predictions were generated, and consider whether the linear-in-year log model overstates late-period growth; a comparison with a more flexible specification (e.g., splines for year) would help demonstrate that the widening-gap conclusion is not an artifact of functional form.

**Response:** The reviewer’s concern regarding the functional form of the adjusted prediction curves was investigated directly. First, the covariate settings used for the original Figure 3 were reconstructed from the analytical workflow. Predictions were generated at age 16 years and at median covariate values of 8 singles competitive event entries, 7 doubles competitive event entries, and 15 total competitive event exposures, respectively, with COVID status set to non-COVID. Under the original linear-in-year log1p specification, the adjusted elite Singles PPE prediction for 2025 was reproduced as 390.03, compared with an observed 2025 elite median of 164.55. This confirmed that the late-period magnitude of the back-transformed trajectory was strongly influenced by the linear-in-year assumption on the log scale.

To assess whether the substantive temporal differentiation between elite and non-elite players depended on this functional form, additional player-year sensitivity models were fitted using natural cubic splines for centred year with three degrees of freedom interacted with elite status. Because the exceptionally deep 2020–2021 source-data coverage could disproportionately influence a flexible temporal function, the COVID-disrupted seasons were excluded from the principal spline sensitivity analysis. The models otherwise retained the same adjustment for age and discipline-specific competitive event exposure, with cluster-robust standard errors at the player level. Joint robust Wald tests of the spline-by-elite-status interaction remained statistically significant for Singles PPE, χ²(3) = 34.79, p < .001, Doubles PPE, χ²(3) = 28.02, p < .001, and Total PPE, χ²(3) = 42.18, p < .001.

The flexible models nevertheless qualified the interpretation of the original linear interaction. The spline trajectories were clearly non-linear, and contrasts comparing the elite–non-elite difference between 2004 and 2025 were not statistically significant for Singles PPE (b = 0.158, 95% CI [−0.052, 0.368], p = .140), Doubles PPE (b = 0.074, 95% CI [−0.249, 0.397], p = .654), or Total PPE (b = 0.152, 95% CI [−0.044, 0.348], p = .128). Accordingly, the substantive interpretation has been refined rather than treated as unchanged: elite and non-elite players exhibited significantly different temporal trajectories, with comparatively pronounced separation in the later part of the observation period, but the data do not support a uniform monotonic widening across the entire 2004–2025 period.

Figure 3 has therefore been replaced by adjusted predictions from the non-COVID natural-spline models. The revised figure displays predictions at age 16 years and at median covariate values of 8 singles competitive event entries, 7 doubles competitive event entries, and 15 total competitive event exposures, with cluster-robust 95% confidence intervals. The original linear interaction estimates have been retained in Table 4 as parsimonious summaries of the average temporal trend, but they are no longer used to represent the literal year-specific shape of the adjusted trajectories. The Methods, Results, Discussion, limitations, Conclusion, and Figure 3 caption have been revised accordingly.

Trend and forecasting models on annual medians. These models are fitted to approximately 20 annual data points per group, and the forecasts inherit this fragility (acknowledged via wide prediction intervals). This is acceptable as a descriptive projection, but please report the number of time points used in each model and temper language accordingly (e.g., Table 5 values reported to two decimals implies more precision than the data support).

**Response:** The number of annual observations used for forecasting has now been stated explicitly. After exclusion of the COVID-disrupted seasons 2020–2021, each forecasting model was fitted to exactly 20 elite annual median observations, corresponding to the years 2004–2019 and 2022–2025. This information has been added to both the Methods and Results. To avoid implying unwarranted numerical precision from these short annual series, all forecasted PPE values, 95% confidence intervals, and 95% prediction intervals in Table 5 have been rounded to one decimal place, and the corresponding values reported in the Results have been revised accordingly. The interpretation has also been further tempered to emphasise that these estimates represent descriptive, conditional continuations of the observed non-COVID historical trend rather than precise or deterministic predictions of future annual values.

Overlap with the author's prior publications. References 2 (Bozděch, 2026, BMC Sports Sci Med Rehabil: "Performance thresholds, efficiency, and access equity in junior girls' tennis: longitudinal analysis and Bayesian forecasting of ITF rankings (2004–2029)") and 19 (Bozděch, 2026, PLOS ONE) appear closely related in dataset, population, and analytical aims. The manuscript briefly contrasts itself with reference 19, but the conceptual and empirical distinction from reference 2 — which also addresses efficiency and forecasting in junior girls' tennis over an almost identical period — should be made explicit in the Introduction so that readers can assess the incremental contribution and the editor can evaluate potential overlapping publication.
**Response:** Thank you for highlighting the need to delineate the present manuscript more explicitly from the two closely related 2026 publications. This relationship has now been described directly in the Introduction. All three studies draw on publicly available ITF World Tennis Tour Juniors ranking archives and therefore share an underlying data source and overlapping calendar years. The previously published PLOS ONE study differs additionally in its male study population, whereas the earlier BMC study and the present manuscript overlap substantially in their female target population and in the 2004–2024 observation period. The principal units of inference, analytical outcomes, and modelling objectives nevertheless differ, as detailed below.

The previously published PLOS ONE study [19] analysed a male junior population over 2004–2024 (n = 8,082) and addressed system-level performance inflation through annual P90, P75, and P50 thresholds for TRP and aggregated PPE, together with developmental and geographic analyses and Bayesian Prophet forecasting through 2029. The BMC Sports Science, Medicine and Rehabilitation study [2] analysed female junior records over 2004–2024 (8,413 player-year observations) and similarly focused on population-level TRP/PPE percentile thresholds, age distributions, geographic representation at P90, and Bayesian Prophet forecasting of threshold trajectories. Thus, the BMC study has the closest population and temporal overlap with the present manuscript.

The present study uses female junior data through 2025 but changes the principal unit of inference from population-level percentile thresholds to within-population differentiation between annually defined elite and non-elite groups. Elite status is derived from an annual structural knee rather than from the P90/P75/P50 categories used in the previous publications. The principal outcomes are separate Singles PPE, Doubles PPE, and Total PPE trajectories, analysed using cluster-robust player-year models and supplemented by year-normalised, COVID-period, lagged, and flexible-year sensitivity analyses. The prospective component also differs from the previous Prophet threshold forecasts: the present study models elite annual median PPE for 2026–2030 and translates forecasted Total PPE into conditional event-exposure scenarios using empirical 2025 TRP benchmarks with propagated bootstrap uncertainty. Although the earlier BMC study included a cross-sectional illustration translating 2024 percentile thresholds into approximate event requirements, the longitudinal scenario analysis and uncertainty-propagation framework reported here were not part of that publication.

Accordingly, the present manuscript does not re-present the previously published percentile-threshold, geographic-equity, or Prophet-forecast results as new findings. Instead, it addresses a distinct question concerning the within-population development of ranking-point production efficiency among structurally defined female player groups. The Introduction has been expanded to state the shared data provenance, temporal overlap, and analytical distinctions explicitly.

In summary, this is a carefully caveated and transparent piece of descriptive ranking-system research with a genuinely useful framing. The classification inconsistency, the unexplained annual sample imbalance and its effect on the pooled descriptives, and the delineation from the author's closely related 2026 publications are the main issues to resolve. I recommend major revision, and I expect the author can address these points without new data collection.

# Reviewer #2:

I believe the manuscript is suitable for publication in PLOS ONE following a minor revision to address some conceptual clarifications and methodological details. The work is commendable for its clarity and potential practical impact on coaching, federation planning, and player development.

While the paper thoroughly analyzes the trends, its practical implications are somewhat tangential. The introduction of "access equity" and the challenges for players with fewer financial resources is mentioned but not directly addressed by the analysis.
**Response:** Thank you for this clarification. The manuscript has been revised to distinguish more explicitly between variables analysed empirically and contextual mechanisms that were not available in the present dataset. Geographic access equity, socioeconomic resources, financial constraints, and differences in federation support were not analysed as outcomes or explanatory variables in the present study, and no conclusions regarding equity are therefore drawn from the results. References to geographical access and financial or federation support have been retained only where they are presented as plausible contextual mechanisms that could influence ranking-point opportunities and PPE. The Discussion and Strengths and limitations sections now state explicitly that these factors were not measured and that the present analyses cannot determine whether the observed ranking-efficiency differences reflect geographic or socioeconomic inequality. The empirical focus of the manuscript remains on ranking-point production efficiency, elite–non-elite temporal differentiation, and conditional event-exposure scenarios.

The forecasting model (linear trend of annual medians) is a simple but effective approach for "a continuation of the observed trend." However, more justification is needed for why a more complex time-series model (e.g., ARIMA) or a model incorporating the uncertainty of the 2025 benchmarks was not considered.

**Response:** The rationale for retaining a simple linear forecasting specification has now been made explicit. After exclusion of the COVID-disrupted seasons 2020–2021, only 20 annual median observations were available for each PPE series. The forecasting objective was not to identify an optimal predictive algorithm, but to provide a transparent and interpretable continuation of the observed non-COVID historical trend. With such short annual series, reliable identification, selection, and validation of autoregressive and moving-average structure would have been limited, while data-driven selection among alternative ARIMA specifications would have introduced additional model-selection uncertainty. The linear model was therefore retained as a parsimonious descriptive specification rather than being presented as superior to alternative time-series approaches. The Methods and Discussion have been revised to state this rationale explicitly, and the forecasts are consistently described as conditional trend-based projections rather than deterministic predictions.

The reviewer’s additional concern regarding uncertainty in the 2025 TRP benchmarks was addressed empirically. The three 2025 reference benchmarks were based on 13 structurally defined elite observations. A paired bootstrap analysis with 10,000 resamples was therefore conducted while holding elite classification fixed. Within each resample, the 2025 TRP P25, median, and P75 and the corresponding elite median Total PPE were recalculated; the resampled 2025 Total PPE median was then incorporated into the 20-observation annual forecasting series, the Total PPE forecast was refitted, and forecast uncertainty was propagated into the required-exposure calculation. The resulting 95% bootstrap intervals for the 2025 TRP benchmarks were 1,830.50–2,407.25 for P25, 1,981.50–2,794.25 for the median, and 2,235.25–2,896.25 for P75. After joint propagation of benchmark and forecast uncertainty, the 2030 required-exposure intervals were 13.02–23.76 for the entry-level benchmark, 14.97–27.05 for the typical benchmark, and 17.34–32.03 for the high benchmark. These intervals were moderately wider than those obtained when only forecast uncertainty was considered, while the scenario point estimates remained unchanged. Figure 5 has also been updated to display the propagated uncertainty intervals across the full 2026–2030 scenario trajectories, allowing the uncertainty around the point estimates and the overlap between benchmark scenarios to be visualised directly. Table 6, the Methods, Results, Figure 5 and its caption, and the limitations have been revised accordingly. The 2025 benchmarks continue to be presented as empirical scenario references rather than normative standards.

The paper is very careful to frame the findings as ranking-system efficiency. However, it could do more to discuss the potential underlying causes for this efficiency gap.

**Response:** Thank you for this suggestion. The Discussion has been expanded to provide a more structured interpretation of the possible mechanisms underlying the observed ranking-efficiency differentiation. Four non-mutually exclusive classes of mechanisms are now considered: ranking-system mechanisms, including changes in point allocation, tournament-grade weighting, and the distribution of ranking-point opportunities; competition-selection mechanisms, including event choice, scheduling, and singles–doubles specialisation; player-performance mechanisms, including match winning, progression within entered events, and unmeasured technical, tactical, physical, or developmental characteristics; and resource- and access-related mechanisms, including geographical access, financial resources, and federation support. These mechanisms are explicitly presented as plausible explanations rather than as causal findings. The revised Discussion also states that the available player-year dataset did not contain tournament grade, round reached, opponent quality, match-level performance, detailed scheduling strategy, or resource-related variables, and therefore did not allow the relative contribution of these mechanisms to be separated. The interpretation consequently remains restricted to ranking-point production efficiency within the historical ITF junior ranking environment.

The author uses both "tournament" and "event" almost interchangeably. For example, "Total event exposure," "tournaments played," and "event entries" are all used. This could be confusing for the reader.

**Response:** Thank you for identifying this terminological ambiguity. The manuscript has been revised globally to distinguish between the analytical unit used in the PPE denominator and a tournament as an organisational competitive unit. Throughout the revised manuscript, competitive event entry denotes a singles or doubles entry contributing to the PPE denominator, competitive event exposure is used as the corresponding general analytical term, and total competitive event exposure denotes the sum of singles and doubles competitive event entries. Because a player may record both a singles and a doubles entry within the same tournament, total competitive event exposure does not necessarily equal the number of unique tournaments entered. The term tournament is now reserved for the underlying organised competition or its characteristics, such as tournament grade, tournament category, or tournament selection. References to “tournament load”, “tournament/event exposure”, “events played”, and deterministic numbers of “tournaments required” have therefore been replaced with the corresponding event-exposure terminology in the Methods, Results, Discussion, Conclusion, table notes, figure captions, and Supporting Information captions.

This is a high-quality, original piece of research that will be of great interest to sports scientists, tennis federations, and coaches. The study is methodologically sound, the findings are robust, and the author's cautious interpretation is a model of scientific integrity. The suggested revisions are minor but would serve to enhance the paper's conceptual clarity and practical impact. I recommend its acceptance after minor revisions.
